# EPCR promotes breast cancer progression by altering SPOCK1/testican 1-mediated 3D growth

**DOI:** 10.1186/s13045-017-0399-x

**Published:** 2017-01-19

**Authors:** Naiara Perurena, Carolina Zandueta, Susana Martínez-Canarias, Haritz Moreno, Silvestre Vicent, Ana S. Almeida, Elisabet Guruceaga, Roger R. Gomis, Marta Santisteban, Mikala Egeblad, José Hermida, Fernando Lecanda

**Affiliations:** 1 Adhesion and Metastasis Laboratory, Program Solid Tumors and Biomarkers, Center for Applied Medical Research (CIMA), University of Navarra, 31008 Pamplona, Spain; 2 Proteomics, Genomics and Bioinformatics Core Facility, Pamplona, Spain; 3 Cardiovascular Sciences Program, Center for Applied Medical Research, University of Navarra, Pamplona, Spain; 4 IdiSNA, Navarra Institute for Health Research, Pamplona, Spain; 5 Department of Histology and Pathology, University of Navarra, Pamplona, Spain; 6 Department of Oncology, Clínica Universidad de Navarra, Pamplona, Spain; 7 Oncology Program, Institute for Research in Biomedicine, Barcelona, Spain; 8 Cold Spring Harbor Laboratory, Cold Spring Harbor, NY, USA

**Keywords:** Matricellular, Metastasis, Microenvironment, Sphere cultures, Extracellular matrix

## Abstract

**Background:**

Activated protein C/endothelial protein C receptor (APC/EPCR) axis is physiologically involved in anticoagulant and cytoprotective activities in endothelial cells. Emerging evidence indicates that EPCR also plays a role in breast stemness and human tumorigenesis. Yet, its contribution to breast cancer progression and metastasis has not been elucidated.

**Methods:**

Transcriptomic status of EPCR was examined in a cohort of 286 breast cancer patients. Cell growth kinetics was evaluated in control and EPCR and SPARC/osteonectin, Cwcv, and kazal-like domains proteoglycan (SPOCK1/testican 1) silenced breast cancer cells in 2D, 3D, and in co-culture conditions. Orthotopic tumor growth and lung and osseous metastases were evaluated in several human and murine xenograft breast cancer models. Tumor-stroma interactions were further studied in vivo by immunohistochemistry and flow cytometry. An EPCR-induced gene signature was identified by microarray analysis.

**Results:**

Analysis of a cohort of breast cancer patients revealed an association of high EPCR levels with adverse clinical outcome. Interestingly, EPCR knockdown did not affect cell growth kinetics in 2D but significantly reduced cell growth in 3D cultures. Using several human and murine xenograft breast cancer models, we showed that EPCR silencing reduced primary tumor growth and secondary outgrowths at metastatic sites, including the skeleton and the lungs. Interestingly, these effects were independent of APC ligand stimulation in vitro and in vivo. Transcriptomic analysis of EPCR-silenced tumors unveiled an effect mediated by matricellular secreted proteoglycan SPOCK1/testican 1. Interestingly, SPOCK1 silencing suppressed in vitro 3D growth. Moreover, SPOCK1 ablation severely decreased orthotopic tumor growth and reduced bone metastatic osteolytic tumors. High SPOCK1 levels were also associated with poor clinical outcome in a subset breast cancer patients. Our results suggest that EPCR through SPOCK1 confers a cell growth advantage in 3D promoting breast tumorigenesis and metastasis.

**Conclusions:**

EPCR represents a clinically relevant factor associated with poor outcome and a novel vulnerability to develop combination therapies for breast cancer patients.

**Electronic supplementary material:**

The online version of this article (doi:10.1186/s13045-017-0399-x) contains supplementary material, which is available to authorized users.

## Background

Endothelial protein C receptor (EPCR) is an endothelial type 1 transmembrane receptor that enhances the activation of protein C (PC) by the thrombin (IIa)-thrombomodulin (TM) complex [1]. EPCR-dissociated activated protein C (APC) negatively regulates the coagulation process, while EPCR-bound APC induces cytoprotective signaling through the proteolytic cleavage of protease-activated receptor 1 (PAR1), leading to anti-inflammatory and anti-apoptotic responses [2].

Recently, research in EPCR has gained considerable momentum by the identification of new EPCR ligands [2]. An EPCR domain distinct from the APC binding site was shown to interact with a specific T cell antigen receptor with potential implications in immunosurveillance of tumors [3]. EPCR was also identified as the endothelial receptor for some subtypes of the erythrocyte membrane protein 1 (PfEMP1) on the surface of the parasite *Plasmodium falciparum*, mediating its sequestration in the blood vessels during severe malaria [4]. FVII/FVIIa has been shown to bind EPCR with a similar affinity as PC/APC [5], whereas the binding of FX/FXa to EPCR remains an open question [6].

Recently, EPCR has been identified as a marker of multipotent mouse mammary stem cells (MaSCs). These EPCR^+^ cells (accounting for 3–7% of basal cells) exhibited a mesenchymal phenotype and enhanced colony-forming abilities [7]. EPCR was also shown to be necessary for cell organization and growth of human mammary epithelial cells in 3D cultures [8].

In cancer, aberrant expression of EPCR is detected in tumors of different origin including the lung [9], breast [10], ovarian [11], colon [12], glioblastoma [13], mesothelioma [14], and leukemia [13]. In lung tumorigenesis, APC/EPCR drives an anti-apoptotic program that endows cancer cells with increased survival ability, enhancing their metastatic activity to the skeleton and adrenal glands [9]. Moreover, high expression levels of this single gene at the primary site in early stage lung cancer patients predict the risk of adverse clinical progression [9, 15].

In breast cancer patients, tumor cells often disseminate to target sites including the skeleton, lungs, brain, and lymph nodes [16]. This event represents a frequent complication associated with a 5-year survival rate ~25.9%. Recent findings have unveiled novel markers in the primary tumor that predict the development of metastasis to target organs such as the skeleton [17]. High EPCR levels have been associated with poor disease progression in the polyoma middle T (PyMT) breast cancer model, closely similar to the luminal B type in humans [18]. Moreover, EPCR^+^ sorted MDA-MB-231 human breast cancer cells showed stem cell-like properties and enhanced tumor-initiating activity, an effect inhibited by APC-EPCR blocking antibodies [18]. In contrast, overexpression of EPCR in MDA-MB-231 cells resulted in reduced final tumor volumes in a xenograft model despite favoring tumor growth at initial stages [19]. The effect of EPCR at different stages of tumor progression remains poorly defined.

In this study, we addressed the functional role of EPCR in primary and metastatic tumor growth in breast cancer using several human and murine xenograft models. We found that EPCR silencing impaired orthotopic tumor growth and metastatic activity to the skeleton and lungs. Moreover, high EPCR expression levels associated with a poor clinical outcome in a cohort of breast cancer patients. Furthermore, we showed that EPCR effects in tumor progression were APC independent and were partially mediated by a novel mechanism involving SPOCK1. Thus, these findings unveil a novel mechanism mediated by EPCR in tumorigenesis and metastasis of breast cancer with potential clinical impact on the therapeutic management of breast cancer patients.

## Methods

### Cell lines and reagents

One thousand eight hundred thirty-three human breast cancer cell line was a kind gift from Dr. Massagué (Memorial Sloan-Kettering Cancer Center, NY, USA) [20]. ANV5 murine breast cancer cell line was previously described [21, 22]. APC (Xigris®) was purchased from Eli Lilly (Indianapolis, IN, USA). Anti-EPCR antibodies RCR252 and RCR1 were kindly provided by Dr. Fukudome (Saga Medical School, Japan) while 1489 was kindly gifted by Dr. Esmon (Oklahoma Medical Research Foundation, Oklahoma City, USA). F(ab´)_2_ fractions of the RCR252 antibody were obtained as previously detailed [9]. shRNAs cloned into PLKO.1-puro vector and the empty vector were obtained from Mission® (Sigma-Aldrich).

### Cell proliferation assay

Cell proliferation was assessed using CellTiter 96® AQueous One Solution Cell Proliferation Assay (MTS), according to manufacturer’s recommendations (Promega). All absorbance values were normalized with the absorbance values from day 0 (5 h after seeding cells).

### Cell cycle analysis

Cell cycle analysis was carried out with Click-iT® EdU Flow Cytometry Assay Kit (Invitrogen). Cells were maintained in culture for 24 or 48 h before adding 10 μM EdU for 2 h. Next, cells were harvested, fixed in formaldehyde (Click-iT® fixative), permeabilized in 1X Click-iT® saponin-based permeabilization and wash reagent, and incubated with the Click-iT® reaction cocktail for 30 min at room temperature in the dark. After a washing step, cells were incubated with 0.2 μg/μl RNase A (Sigma-Aldrich) for 1 h at room temperature, in the dark. 7AAD was added to the tubes 10 min before the acquisition of cells in a FACSCanto II cytometer (BD Biosciences). Data were analyzed using FlowJo® software v9.3.

### Annexin-V flow cytometry assay

Cells were seeded into 24-well plates and cultured for 24 h. Next, cells were incubated with 2 μM staurosporine for 1 h or serum-starved overnight before the addition of 50 nM APC for 4 h followed by 2 μM staurosporine for 1 h next day. After staurosporine treatment, cells were harvested, resuspended in annexin-binding buffer (10 mM HEPES, 140 mM NaCl, and 2.5 mM CaCl_2_, pH 7.4) and incubated with Alexa Fluor 647-conjugated annexin-V and 7AAD (BD Biosciences) for 15 min at room temperature, in the dark. Cells were acquired in a FACSCanto II cytometer (BD Biosciences) and analyzed using FlowJo® software v9.3.

### Cell culture in 3D

Culture media was mixed at 1:1 ratio with Growth Factor Reduced Matrigel (BD Biosciences). One hundred microliters of the mix were added to each well of a 96-well plate and incubated at 37 °C for 30 min. Five hundred (1833, BT-549, ANV5, MCF10A) or 1000 (MDA-MB-231) cells in medium with 10% matrigel were added on top of the coating and maintained in culture for 8–10 days. Medium with 10% matrigel was replaced at day 4–5. Pictures of the spheres were taken at day 8–10 at ×4 magnification using an inverted microscope (Leica) and analyzed using Fiji software [23].

### In vivo experiments

Athymic nude mice (*Foxn1* ^*nu*^) were purchased from Harlan (Barcelona, Spain) and maintained under specific pathogen-free conditions. Five- to six-week-old mice were used for all experiments. RAG-2^−/−^ mice were bred at the in-house Animal Core Facility and used for the intratibial experiment. For the orthotopic injection, 50 μl containing 500,000 cells resuspended in Growth Factor Reduced Matrigel (BD Biosciences) mixed with PBS at 1:1 ratio were directly injected into the fourth mammary fat pads of mice (2 tumors per mouse). In the second orthotopic experiment, cells were injected resuspended in 20 μl of PBS without matrigel. Tumor growth was monitored regularly using a digital caliper and tumor volume was calculated as follows: *π* × length × width^2^/6. For intracardiac injection, 10^5^ cells in 100 μl of PBS were inoculated into the left cardiac ventricle, using a 29G needle syringe [24]. For intratibial injection, 15,000 cells in 5 μl of PBS were injected into the tibia’s bone marrow through the femoro-tibial cartilage using a Hamilton syringe [25]. For intravenous injection, 100,000 cells in 100 μl of PBS were injected through the tail vein of mice. For BLI, animals were anesthetized and inoculated with 50 μl of 15 mg/ml D-luciferin (Promega). Images were taken during 1 min with a PhotonIMAGER™ imaging system (Biospace Lab) and analyzed using M3Vision software (Biospace Lab). Photon flux was calculated by using a region of interest (ROI) or by delineating the mouse for whole-body bioluminescence quantification. All bioluminescence signals were normalized with values from day 0, except for the metastasis experiment with RCR252 treatment. Radiographic and micro-computed tomography (Micro-CT) analyses were performed as described elsewhere [26].

### Microarray analysis

RNA was extracted from snap-frozen mammary tumors and hybridized to Human Gene ST 2.0 microarrays (Affymetrix). Data were normalized with RMA (Robust Multi-Array Average) approach. Low expression probes were removed by filtering those that did not exceed a level of expression of 32 in at least one of the samples for each condition. Differentially expressed genes were identified using LIMMA (linear models for microarray data) method [27].

### Statistical analysis

Statistical analysis was performed using SPSS v15.0. When data exhibited homoscedasticity, pairwise Student’s *t* test and Mann–Whitney *U* test were used for normally and non-normally distributed variables, respectively. When data exhibited heterocedasticity, Welch and Median tests were used for normally and non-normally distributed variables, respectively. ANOVA and posterior Bonferroni tests were used for multiple comparisons of normally distributed variables. Kruskal–Wallis and posterior Bonferroni adjusted-Mann–Whitney *U* tests were used for multiple comparisons of non-normally distributed variables. Statistical significance was defined as significant (*p* < 0.05, *), very significant (*p* <0.01, **) and highly significant (*p* < 0.001, ***). Other additional methods are included in the Additional file 1.

## Results

### High EPCR expression in breast tumors correlates with poor clinical outcome

**Fig. 1:**
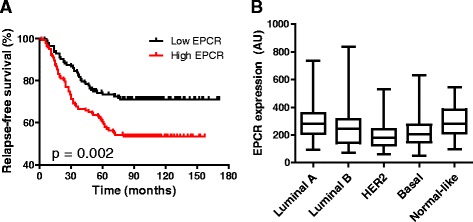
Kaplan–Meier analysis in breast cancer patients based on EPCR expression levels. **a** Relapse-free survival analysis of all patients included in the GSE2034 cohort (*n* = 286), classified into “high EPCR” and “low EPCR” based on median expression value of EPCR. **b** EPCR expression levels in the primary tumors, classified by molecular subtype. Whiskers represent minimum and maximum values. *AU*, arbitrary units

To evaluate the association between EPCR expression levels and risk of metastasis in breast cancer, we performed a relapse-free survival analysis in a cohort of 286 patients, including 106 patients with distant relapses (GSE2034) [28]. EPCR expression levels were classified as “high” or “low” according to the median. We found that patients with high EPCR expression levels had significantly shorter relapse-free survival times (Fig. 1a) (Additional file 2: Figure S1). The clinical predictive potential of EPCR was not related to a higher EPCR expression in different molecular subtypes (Fig. 1b). Overall, these results indicate that EPCR is a poor prognosis factor in breast cancer patients.

### EPCR silencing impairs breast tumorigenesis and spontaneous metastases

**Fig. 2:**
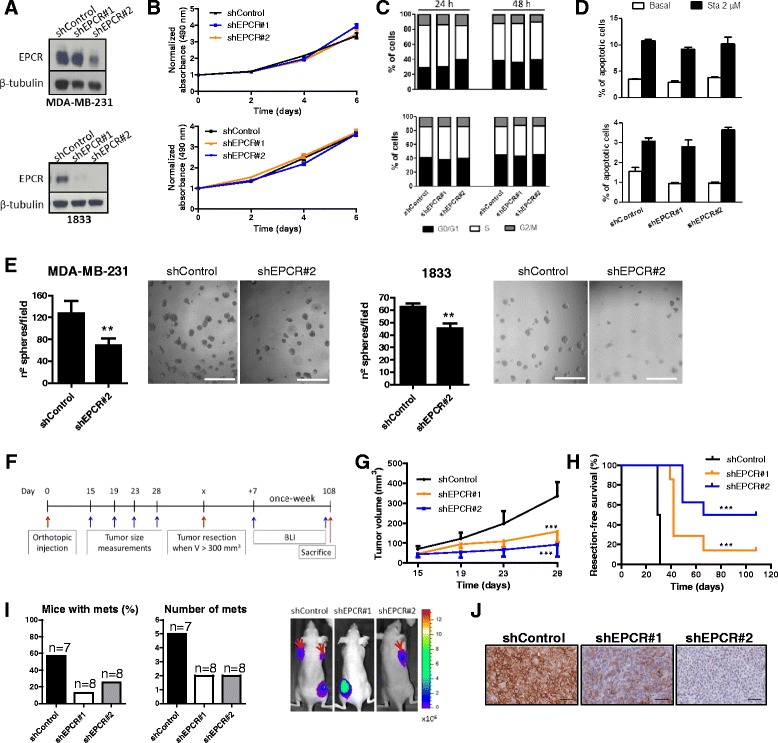
Effects of EPCR silencing in vitro and in vivo tumor growth in an orthotopic xenograft model. **a** Western blot analysis of EPCR protein levels in MDA-MB-231 and 1833 cells transduced with a scramble shRNA (shControl) and two different shRNAs targeting human EPCR (shEPCR#1 and shEPCR#2). β-tubulin was used as loading control. **b** MTS in vitro proliferation assay of MDA-MB-231 (*top*) and 1833 (*bottom*) cells. Data were normalized with absorbance values from day 0 and represent mean ± SD of six replicates. Experiments were repeated three times with similar results. **c** Percentage of MDA-MB-231 (*top*) and 1833 (*bottom*) cells in each phase of the cell cycle, after maintaining cells in culture for 24 and 48 h. **d** Percentage of apoptotic MDA-MB-231 (*top*) and 1833 (*bottom*) cells in basal and staurosporine-induced conditions, measured by annexin-V binding flow cytometry assay. Data are mean ± SD of triplicates and representative of three independent experiments. *Sta* staurosporine **e** Quantification of spheres grown in 3D matrigel cultures. Data are mean ± SD of 8 replicates from two independent experiments. Representative images at ×4 magnification. *Scale bar* 0.5 mm. **f** Outline of the in vivo orthotopic experiment (*n* = 8 per group). **g** Quantification of tumor volume until day 28 post-injection. *Each dot* represents mean ± SEM. **h** Kaplan–Meier curves of resection-free survival. **I** Incidence of metastatic events and representative images showing metastases (*red arrows*), assessed by BLI. *Mets* metastases. **j** Representative images at ×20 magnification showing immunohistochemical staining of EPCR in formaldehyde-fixed mammary tumors. *Scale bar* 50 μm. **p < 0.01, ***p < 0.001

To study the role of EPCR, we used several triple-negative breast cancer cell lines, including human cell lines (MDA-MB-231 and its bone metastatic derivative 1833, and BT-549) and the ANV5 murine cell line. We silenced EPCR expression levels by lentiviral transduction of different shRNAs targeting human (shEPCR#1 and shEPCR#2) and murine (shEPCR#3) EPCR and a scramble shRNA (shControl) as control (Fig. 2a and Additional file 3: Figure S2A). EPCR knockdown did not affect cell proliferation, cell cycle progression, or basal and induced apoptosis of MDA-MB-231, 1833, BT-549, and ANV5 cells in 2D cultures (Fig. 2b–d and Additional file 3: Figure S2A–D). However, EPCR knockdown significantly reduced the number of spheres grown in 3D matrigel cultures in all cell lines tested (Fig. 2e and Additional file 3: Figure S2E).

To explore the relevance of these findings in vivo, we performed an orthotopic experiment using the highly metastatic subpopulation 1833, as outlined in Fig. 2f. Remarkably, EPCR silencing significantly reduced primary tumor growth after the injection of shControl, shEPCR#1, or shEPCR#2 1833 cells into the fourth mammary fat pads of athymic nude mice, in two independent experiments (Fig. 2g). Consistently, time until resection of tumors at 300 mm^3^ was significantly longer for EPCR-silenced groups, showing that control tumors maintained higher proliferation rates over the course of the experiment (Fig. 2h). Of note, several tumors in EPCR-silenced groups did not reach the size established for tumor resection by the end of the experimental period (Fig. 2h). In addition, BLI performed after tumor resection showed that the number of mice with metastasis and the number of metastatic events were lower in EPCR-silenced groups (Fig. 2i). Importantly, EPCR inhibition was confirmed by immunohistochemistry in resected primary tumors (Fig. 2j). Similarly, in another xenograft model of murine ANV5 cells, EPCR silencing reduced primary tumor growth after orthotopic injection of shControl and shEPCR#3 cells into athymic nude mice. Evaluation of spontaneous metastases in this model was limited by the highly frequent local recurrence after tumor resection (Additional file 3: Figure S2F, G).

Analysis of tumors in the orthotopic model of 1833 cells, either size-matched tumors resected at different time points (Additional file 4: Figure S3) or tumors of different size resected at the same time point (Additional file 5: Figure S4) revealed a slight increase in apoptosis (cleaved caspase-3) and/or necrosis and a lower proliferation rate, assessed by Ki67 staining, in EPCR-silenced tumors. Of note, we did not observe relevant changes in angiogenesis and immune infiltration patterns of tumors (Additional file 5: Figure S4 and Additional file 6: Figure S5). Taken together, these data indicate that EPCR contributes to primary tumor growth and the development of spontaneous metastases in breast cancer.

### EPCR silencing reduces metastasis to the bone and lungs

**Fig. 3:**
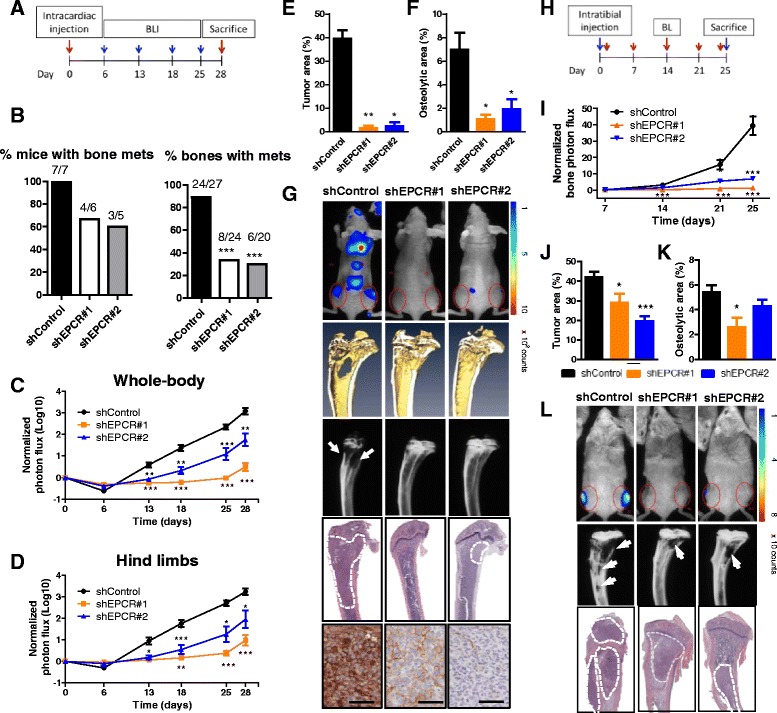
Evaluation of the prometastatic activity of 1833 control and EPCR-silenced cells. **a** Outline of the experiment after intracardiac inoculation (*n* = 8, *n* = 7, and *n* = 6 for shControl, shEPCR#1, and shEPCR#2, respectively). **b** Number of mice and the bones with metastasis in each group. Whole-body (**c**) and hind limbs (**d**) photon flux quantification along the experiment. Data were normalized with BLI values from day 0. **e** Tumor area quantification in H&E-stained bone sections. **f** Osteolytic bone area quantification in X-ray images at day 28 post-injection. **g** Representative images of BLI, micro-CT scans, X-ray scans, H&E-stained bone sections, and immunohistochemical staining of EPCR in tumors, from *top* to *bottom*, respectively. *Scale bar* 20 μm. **h** Outline of the experiment of intratibial bone colonization (*n* = 8 per group). **i** BLI quantification in the hind limbs along the whole experimental period. Data were normalized with BLI values from day 0. **j** Tumor area quantification in H&E-stained bone sections. **k** Osteolytic bone area quantification in X-ray images at day 25 post-injection. **l** Representative images of BLI, X-ray scans, and H&E-stained bone sections, from *top* to *bottom*, respectively. All data represented are mean ± SEM. *p < 0.05; **p < 0.01; ***p < 0.001

Next, we studied the activity of EPCR in additional experimental models of metastasis. The effect of EPCR silencing in bone metastasis was assessed after intracardiac inoculation of shControl, shEPCR#1, and shEPCR#2 1833 cells into athymic nude mice (Fig. 3a). The percentage of mice and the bones with metastases was significantly lower in EPCR-silenced groups (Fig. 3b), consistent with the reduced whole-body and hind limb bioluminescence signals (Fig. 3c, d). Differences in BLI were statistically significant from day 13 of the experiment, suggesting that EPCR promotes tumor growth of cancer cells once they have reached the target organ. Accordingly, EPCR silencing significantly reduced bone tumor burden and the extension of osteolytic lesions at day 28 post-injection (Fig. 3e–g). Importantly, EPCR inhibition by shRNAs was maintained until the end of the experimental period (Fig. 3g, bottom panel). These results substantiate the role of EPCR in breast cancer and indicate that EPCR promotes metastatic activity to the bone. Moreover, the lower incidence of metastatic events in mice injected with EPCR-silenced cells suggests that EPCR is required during metastatic tumor re-initiation at the secondary site.

To further explore the function of EPCR in bone colonization, shControl, shEPCR#1, and shEPCR#2 1833 cells were injected into the tibiae of immunocompromised mice. Bone colonization was analyzed by BLI, X-rays, and histological analysis (Fig. 3h). Tumors developed in all tibiae in shControl and shEPCR#2 mice, while two tibiae remained tumor-free in shEPCR#1 group. Differences in BLI became very relevant at advanced experimental time points (Fig. 3i, l). In addition, histological and X-ray analyses revealed reduced tumor burden and osteolytic bone areas in EPCR-knockdown groups at the end of the experiment (Fig. 3j–l). Of note, the magnitude of the effect of EPCR silencing revealed by different techniques (BLI vs. histology and X-rays) differs, an event probably related to the fact that X-ray analysis does not detect extraosseous tumor grown through the cortical bone on the periosteal surface (Fig. 3l, bottom panel). Similarly, extracortical tumor cells that contribute to tumor burden were lost during histological processing, whereas these cells contribute to BLI. Thus, these results indicate that EPCR promotes metastatic tumor growth in the osseous compartment.

Next, we evaluated the prometastatic activity of EPCR in an intra-tail injection model. For this purpose, we injected shControl and shEPCR#3 ANV5 cells intravenously into athymic nude mice and analyzed lung metastases at the end of the experiment (Additional file 7: Figure S6A). EPCR knockdown was able to block the development of lung metastases, assessed by BLI (Additional file 7: Fig. S6B, D) and tumor area quantification in H&E-stained lung sections (Additional file 7: Figure S6C, D).

### APC does not mediate effects of EPCR silencing in vitro and in vivo

Next, we explored the mechanistic insights of EPCR function in tumor growth and metastasis. First, we analyzed whether the main known ligand of EPCR, APC, could signal and mediate cellular functions to favor tumor progression in MDA-MB-231, 1833, BT-549, and ANV5 cells. Stimulation of cells with APC did not affect their proliferation, cell cycle progression, and resistance to basal and induced apoptosis (Additional file 8: Figure S7). Accordingly, treatment with the F(ab)2´fraction of RCR252 antibody, which blocks APC binding to human EPCR (Additional file 9: Figure S8A), did not reduce bone metastasis of 1833 cells inoculated into the left cardiac ventricle of athymic nude mice (Additional file 9: Figure S8B–F).

### Identification of SPOCK1 as a mediator of EPCR effects

**Fig. 4:**
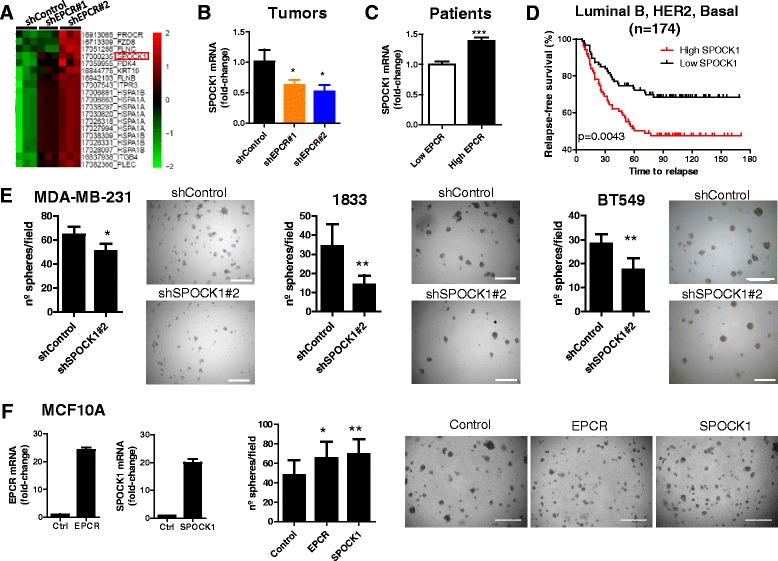
Identification and validation of SPOCK1 as an EPCR effector. **a** Microarray analysis of mammary tumors developed in mice injected with 1833 cells. *Heat map* showing most relevant genes downregulated in both shEPCR#1 and shEPCR#2 tumors compared to shControl tumors. **b** Validation by RT-qPCR of SPOCK1 downregulation in subcutaneous xenograft tumors (1833 cells). Data are mean ± SEM of three tumors. **c** Expression levels of SPOCK1 in breast tumors from patients with low and high EPCR levels, classified by median expression value of EPCR (GSE2034 database). **d** Relapse-free survival analysis in patients from luminal B, HER2+, and basal subsets (GSE2034 database), classified into “high SPOCK1” and “low SPOCK1” based on the median expression value of SPOCK1. Log-rank test was used. **e** Quantification of spheres grown in 3D matrigel cultures. Data are mean ± SD of 8 replicates from two independent experiments. **f** Quantification of the number of spheres grown in 3D matrigel cultures in MCF10A cells overexpressing EPCR or SPOCK1. Data are mean ± SD of 8 replicates from two independent experiments. Representative images at ×4 magnification. *Scale bar* 0.5 mm. *p < 0.05; **p < 0.01; ***p < 0.001

In order to explore other mechanisms mediating EPCR effects, we interrogated Human Gene 2.0 ST microarrays (Affymetrix) to discriminate genes associated with EPCR silencing in size-matched mammary tumors grown in athymic nude mice after orthotopic implantation of shControl, shEPCR#1, and shEPCR#2 1833 cells. An unsupervised clustering analysis revealed several genes related to tumor progression to be downregulated in both EPCR-silenced tumor groups (Fig. 4a). Among these genes, SPOCK1/testican 1, a member of the SPARC family of matricellular proteins, was also downregulated in subcutaneous tumors derived from shEPCR#1 and shEPCR#2 1833 cells, compared to control tumors (Fig. 4b). Moreover, breast cancer patients (GSE2034 cohort) with high EPCR expression also had significantly higher SPOCK1 expression levels (Fig. 4c). Importantly, high SPOCK1 expression levels associated with a significantly shorter relapse-free survival time in patients with luminal B, basal and HER2+ tumors (Fig. 4d) but not luminal A (Additional file 10: Figure S9). Interestingly, these data are consistent with the predictive potential of EPCR levels in these three subsets, but not in luminal A. This finding suggests that EPCR could mediate tumor progression in part by upregulating SPOCK1.

Next, we tested the effects of SPOCK1 in vitro, by silencing SPOCK1 expression levels with shRNAs in MDA-MB-231, 1833, and BT549 human cell lines (Additional file 11: Figure S10A). Interestingly, SPOCK1 silencing did not affect cell growth kinetics in 2D cultures (Additional file 11: Figure S10B, C), but significantly reduced the number of spheres in 3D matrigel cultures in all cell lines (Fig. 4e). Conversely, ectopic expression of EPCR and SPOCK1 in non-tumorigenic MCF10A mammary cells significantly increased the number of spheres in 3D cultures (Fig. 4f). These data indicate that EPCR or SPOCK1 overexpression confers a growth advantage in 3D cultures in a non-tumorigenic mammary cell line, but per se EPCR or SPOCK1 are not sufficient to confer a tumorigenic phenotype requiring an oncogenic background. Taken together, these findings support the role of SPOCK1 mediating EPCR effects and suggest that EPCR could promote 3D growth of breast cancer cells by altering tumor-matrix interactions by modulating SPOCK1.

### SPOCK1 silencing impairs breast tumorigenesis and metastases

**Fig. 5:**
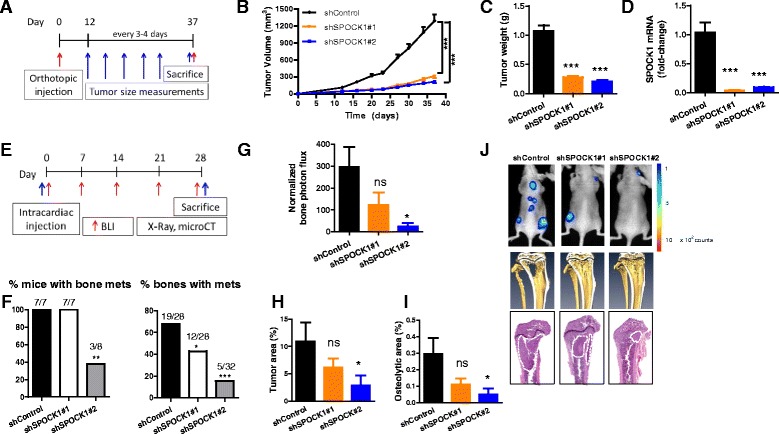
Effects of SPOCK1 silencing in primary tumor growth and metastasis in 1833-derived xenograft models**. a** Outline of the orthotopic experiment to assess primary tumor growth. **b** Tumor volume measurements along the whole experimental period. **c** Weight of resected tumors at day 37 post-injection. **d** mRNA levels of SPOCK1 in resected tumors (*n* = 3 per group), assessed by RT-qPCR. **e** Outline of the intracardiac inoculation experiment to assess bone metastasis. **f** Number of mice and bones with metastases in each group. **g** BLI quantification in hind limbs at day 28 post-injection. **h** Tumor area quantification in H&E-stained bone sections. **i** Osteolytic bone area quantification in micro-CT scans from day 28 post-injection. **j** Representative images of BLI, micro-CT scans, and H&E-stained bone sections, from *top* to *bottom*, respectively. All data are mean ± SEM. *p < 0.05; **p < 0.01; ***p < 0.001. *ns*, non-statistical significance

Next, we explored the role of SPOCK1 in breast tumorigenesis using the previously described orthotopic model. ShControl, shSPOCK#1, or shSPOCK#2 1833 cells were injected into the fourth mammary fat pads of ahtymic nude mice, and tumor growth was evaluated (Fig. 5a). SPOCK1 silencing resulted in a significant reduction in tumor growth (Fig. 5b, c). Importantly, SPOCK1 inhibition by shRNAs was maintained along the whole experimental period (Fig. 5d). Taken together, these results indicate that SPOCK1 is a relevant factor for primary tumor growth in breast cancer.

Finally, we evaluated the bone metastatic activity of control and SPOCK1-silenced 1833 cells after intracardiac inoculation into athymic nude mice (Fig. 5e). All mice in the shControl and shSPOCK1#1 groups developed bone metastases, but only 3 mice in the shSPOCK1#2 group. Moreover, the number of bones with metastases was significantly lower in both SPOCK1-silenced groups compared to shControl (Fig. 5f). Consistently, BLI and H&E staining revealed a lower tumor burden in SPOCK1-silenced groups, associated with a lower osteolytic area (Fig. 5g–j). These results indicate that SPOCK1 silencing recapitulates the effects observed by EPCR silencing in vivo and further support the role of SPOCK1 as an effector of EPCR.

## Discussion

In this work, we unveiled a novel molecular mechanism of EPCR contributing to breast cancer progression favoring tumor growth and metastatic activity to target organs. EPCR endowed cells with advantageous growth in 3D, an effect partially mediated by the extracellular matrix proteoglycan SPOCK1. These cell functions were correlated with increased metastatic risk and poor clinical outcome in breast cancer patients. Importantly, this association was relevant in all the molecular subtypes, except luminal A, indicating that EPCR could be useful as a potential prognostic marker in these patient subsets.

Previous studies identified EPCR as a marker of human breast cancer stem cells with enhanced tumor-initiating and growth abilities in immunodeficient mice [18]. In addition, EPCR deficiency attenuated spontaneous tumor growth in the PyMT murine breast cancer model [18]. In agreement with these findings, we showed that EPCR silencing impaired orthotopic tumor growth of highly metastatic 1833 cells. In this model, differences in tumor size between EPCR^+^ and EPCR^−^ tumors became more relevant at advanced experimental time points. In contrast in another study, although EPCR overexpression increased initial orthotopic growth of MDA-MB-231 cells, it resulted in smaller final tumor volumes [19], a finding possibly related to EPCR loss in evolving tumors. Thus, EPCR could display different roles at different stages of breast cancer progression such as initiation, maintenance, and target organ colonization. Future experiments will help to characterize its role in each of these stages in different histological subtypes.

Besides its role in tumorigenesis, EPCR also displayed a marked prometastatic activity to target organs, events that cooperatively support its contribution to prognosis. The consistent results obtained in both metastatic models indicate that EPCR confers a functional advantage at late stages of the metastatic process. Moreover, differences in metastatic tumor burden became more relevant at advanced experimental time points, indicating an effect more pronounced during the colonization of target organs, as evidenced by the overt osseous colonization observed in the intratibial model.

In contrast with previous findings in lung cancer [9], EPCR did not markedly contribute to tumor cell survival in the circulation and engraftment in secondary sites. The prominent effect in breast cancer during colonization was associated with its role in 3D growth and based on the low number of tumor nodules in shEPCR mice in both models (the bone and lung) of experimental metastasis, EPCR may also modulate metastatic tumor re-initiation at the target organ.

Tumors are organ-like structures composed of tumor cells and stromal cells embedded in a complex ECM within the tumor microenvironment [29]. Components of the ECM such as tenascin C have been shown to promote breast cancer progression and metastasis [30–32]. In the same line, our study identified SPOCK1, a secreted matricellular protein as a markedly downregulated gene in EPCR-silenced tumors. SPOCK1 belongs to the Ca^2+^-binding proteoglycan family which includes SPARC, a well-studied tumor-associated component involved in regulating adhesion, matrix cellular interactions, and cell growth [33, 34]. Recently, SPOCK1 has been shown to promote epithelial-mesenchymal transition (EMT) and metastasis in other tumors including lung and gallbladder cancer and hepatocarcinoma [35–37]. Interestingly, high SPOCK1 expression levels were associated with adverse clinical outcome in the same subsets of breast cancer patients predicted by EPCR levels. Therefore, EPCR could promote tumor growth in vivo, in part, by modulating tumor-matrix interactions through SPOCK1 favoring an advantageous 3D growth of tumorigenic cells. Indeed, SPOCK1 silencing in breast cancer cells impaired the number of 3D spheres and primary and metastatic tumor growth, an effect that phenocopied EPCR silencing. Accordingly, EPCR has been required for cell organization and growth of mammary epithelial cells in 3D cultures [8] In agreement with these findings, EPCR/SPOCK1 axis activation in non-tumorigenic mammalian cells increased the number of spheres grown in 3D matrigel cultures. However, it was not sufficient to confer a tumorigenic phenotype.

A surprising finding of our study was the lack of effects mediated by APC, despite the fact that anti-EPCR blocking antibodies (1535) reduced orthotopic growth of MDA-MB-231 cells in previous studies [18]. Although, we did not specifically address APC/EPCR effects in orthotopic tumors, we explored its contribution in vitro and during the development of bone metastases. EPCR-blocking antibodies in this model could not reduce the metastatic activity of 1833 cells, suggesting that EPCR triggered APC-independent effects. In this experiment, we used the F(ab´)_2_ fractions of the anti-EPCR blocking antibody to avoid any interference of the activated complement system, whereas Schaffner et al. [18] employed whole-body antibodies. Furthermore, the use of the same strategy of F(ab´)_2_ fractions showed a significant effect on a model of lung cancer metastasis underscoring the validity of this approach [9]. Complementary to this view, other ligands different than APC binding to different regions of EPCR in each tumor type or accessible in specific microenvironments could account for these differences. Based on these findings, future experiments should address other mechanisms that could be mediated by EPCR in different tumor types and metastatic sites.

## Conclusions

In summary, our study unveils a novel role of EPCR as a clinically relevant factor in breast cancer, which promotes primary tumor growth and metastatic activities in target organs. Unexpectedly, EPCR modulates tumor cell-ECM interactions involved in 3D growth required for tumor progression and metastasis, in part by upregulating SPOCK1. These findings underscore a novel role of EPCR as a novel prognostic factor and a potential therapeutic target in a subset of breast cancer patients.

## Additional files

Additional file 1: Supplementary material and methods. (DOCX 38.8 kb)

Additional file 2: Figure S1. Kaplan–Meier analysis in different molecular subtypes of breast cancer patients based on EPCR expression levels. Relapse-free survival curves for each molecular subtype of breast cancer. Log-rank test was used to determine *p* values in all cases. (PPTX 273 kb)

Additional file 3: Figure S2. Effects of EPCR silencing in vitro and in vivo tumor growth in an orthotopic model. A. Western blot analysis of EPCR protein levels in human BT-549 (top) and murine ANV5 (bottom) cells transduced with a scramble shRNA (shControl) and shRNAs targeting human (shEPCR#1 and shEPCR#2 in BT-549) and murine (shEPCR#3 in ANV5) EPCR. β-tubulin was used as loading control. White line indicates that the membrane was cut. B. MTS in vitro proliferation assay of BT-549 (top) and ANV5 (bottom) cells. Data were normalized with absorbance values from day 0 and represent mean ± SD of six replicates. C. Percentage of BT549 (top) and ANV5 (bottom) cells in each phase of the cell cycle after maintaining cells in culture for 24 and 48 h. Sta, staurosporine. D. Percentage of apoptotic BT-549 (top) and ANV5 (bottom) cells in basal and staurosporine-induced conditions, measured by annexin-V binding flow cytometry assay. E. Quantification of spheres grown in 3D matrigel cultures. Data are mean ± SD of 8 replicates. Representative images at ×4 magnification. Scale bar 0.5 mm. F. Outline of the in vivo orthotopic experiment (*n* = 8 per group). G. Quantification of tumor volume at day 15 post-injection. H. Kaplan–Meier curves of resection-free survival. (PPTX 9850 kb)

Additional file 4: Figure S3. Immunohistochemical analysis of several markers in control and EPCR-silenced size-matched mammary tumors resected at different time points. A. Representative images showing H&E staining (×2.5 magnification) and the immunohistochemical staining of Ki67, cleaved caspase-3, CD31, and F4/80 (×20 magnification) in formaldehyde-fixed tumors. Scale bars 80 μm (H&E) and 10 μm (Ki67, caspase-3, CD31, and F4/80). T. mass, tumor mass. T. border, tumor border. B. Quantification of the percentage of immunoreactive cells. Each dot represents one tumor. Data are mean ± SEM. ns means non-statistical significance. (PPTX 2780 kb)

Additional file 5: Figure S4. Effects of EPCR silencing in cell growth kinetics and immune infiltration of control and EPCR-silenced mammary tumors resected at the same time point. A. Outline of the experiment (*n* = 5 per group). B. Tumor volume at the end of the experimental period (day 32 post-injection). Each dot represents one tumor. Data are mean ± SEM. C. Representative images showing H&E staining (×2.5 magnification) and the immunohistochemical staining of Ki67, cleaved caspase-3, CD31, and F4/80 (×20 magnification). T. mass, tumor mass. T. border, tumor border. Scale bars 80 μm (H&E) and 10 μm (Ki67, caspase-3, CD31, and F4/80). D. Quantification of the percentage of immunoreactive cells. Each dot represents one tumor. Data are mean ± SEM. *p < 0.05. ns means non-statistical significance. (PPTX 2260 kb)

Additional file 6: Figure S5. Analysis of immune cells infiltrating control and EPCR-silenced mammary tumors. A. Flow cytometry gating strategy. Arrows of the same color indicate simultaneous detection of markers. MDSCs, myeloid derived suppressor cells. NK, natural killer cells. DCs, dendritic cells. B. Quantification of the percentage of immune subpopulations infiltrating the tumors. Each dot represents one tumor. Data are mean ± SEM. (PPTX 932 kb)

Additional file 7: Figure S6. Effects of EPCR silencing in the ability of murine ANV5 cells to metastatize to the lungs. A. Outline of the intra-tail injection experiment (*n* = 8 per group). Quantification of bioluminescence signals (B) and tumor area (C) in the lungs at the end of the experimental period (day 28 post-injection). Each dot represents one mouse. D. Representative images of H&E-stained lung sections (top) and BLI (bottom). *p < 0.05, **p < 0.01. (PPTX 1080 kb)

Additional file 8: Figure S7. Cell growth kinetics of APC-stimulated breast cancer cell lines. A. MTS proliferation assay of cells stimulated with increasing doses of APC. Data were normalized with absorbance values from day 0. Each dot represents mean ± SD of six replicates. B. Percentage of cells in each phase of the cell cycle in control and 50 nM APC-stimulated cells for 24 and 48 h, in serum-free and 4% serum medium. C. Percentage of apoptotic cells in basal and staurosporine-induced conditions, measured by annexin-V binding flow cytometry assay. Cell lines are MDA-MB-231,1833, BT-549, and ANV5, from the left to the right, in all figure sections. (PPTX 325 kb)

Additional file 9: Figure S8. Effects of the pharmacological EPCR blockade in the prometastatic activity of 1833 cells. A. Specificity of anti-EPCR antibodies, RCR252, and its F(ab´)2 fraction, by surface plasmon resonance (SPR). EPCR (500 RU) was immobilized through the anti-EPCR antibody RCR2 (that does not bind in the ligand-receptor domain) on a CM5 chip. The binding of 250 nM of RCR252 and its F(ab´)2 fraction to the EPCR were monitored. A representative experiment is shown. RU resonance units; s, seconds. B. Outline of the experiment (*n* = 8 per group). C. Photon flux quantification in hind limbs. D. Tumor area quantification in H&E-stained bone sections. E. Osteolytic bone area quantification in X-ray images from day 28 post-injection. F. Representative images of BLI (top), X-rays (middle), and H&E staining (bottom) at day 28 post-injection. All data are represented by mean ± SEM. (PPTX 1540 kb)

Additional file 10: Figure S9. Clinical relevance of SPOCK1 in different breast cancer subtypes. A. Relapse-free survival analysis of all patients included in the GSE2034 cohort (*n* = 286), classified into “high SPOCK1” and “low SPOCK1” based on median expression value of SPOCK1. B. SPOCK1 mRNA expression levels in the primary tumors, classified by molecular subtypes. Whiskers represent minimum and maximum values. AU, arbitrary units. C. Relapse-free survival curves for each molecular subtype of breast cancer. Log-rank test was used to determine *p* values in all cases. ns, non-statistical significance. (PPTX 288 kb)

Additional file 11: Figure S10. Cell growth kinetics in 2D cultures of human breast cancer cell lines after SPOCK1 silencing. A. Analysis of SPOCK1 expression levels by RT-qPCR in human cells transduced with a scramble shRNA (shControl) and two different shRNAs (shSPOCK#1 and shSPOCK#2) targeting human SPOCK1. B. MTS in vitro proliferation assay of MDA-MB-231 (top), 1833 (middle), and BT549 (bottom) cells. Data were normalized with absorbance values from day 0 and represent mean ± SD of six replicates. Experiments were repeated three times with similar results. C. Percentage of MDA-MB-231 (top), 1833 (middle), and BT549 (bottom) cells in each phase of the cell cycle, after maintaining cells in culture for 24 and 48 h. (PPTX 223 kb)

## Abbreviations

3D: 3-dimension
APC/EPCR: Activated protein C/endothelial protein C receptor
BLI: Bioluminescence imaging
MaSCs: Mammary stem cells
PAR1: Protease-activated receptor 1
PfEMP1: Erythrocyte membrane protein 1
PyMT: Polyoma middle T
SPOCK1: SPARC/osteonectin, Cwcv and kazal-like domains proteoglycan
