# Supplementary material for: EPCR promotes breast cancer progression by altering SPOCK1/testican 1-mediated 3D growth
Source: J Hematol Oncol. 2017 Jan 19;10:23. doi: 10.1186/s13045-017-0399-x (PMC5248526; doi:10.1186/s13045-017-0399-x)
Supplement: Supplementary file 1 — Supplementary material and methods. (DOCX 38.8 kb) [file 13045_2017_399_MOESM1_ESM.docx]

**EPCR PROMOTES BREAST CANCER PROGRESSION BY ALTERING SPOCK1-MEDIATED 3D GROWTH**

**Perurena N et al.**

**SUPPLEMENTARY MATERIAL AND METHODS**

**Cell lines and reagents**

MDA-MB-231 and 1833 cell lines were cultured in Dulbecco’s Modified Eagle’s Medium (DMEM) (Sigma-Aldrich). BT549 cells were cultured in RPMI 1640 (Lonza). ANV5 cell line was cultured in RPMI 1640 (Lonza) supplemented with 10 mM HEPES (Lonza), 1% GlutaMAX™ (GIBCO) and 1 mM sodium pyruvate (Lonza). All media were supplemented with 10% heat-inactivated (30’ at 56°C) fetal bovine serum (FBS) (GIBCO), 100 units/ml penicillin and 100 µg/ml streptomycin (GIBCO), referred to as complete medium.

MCF10A cell line (kind gift of C. Berasain, CIMA) was cultured according to standard protocols (<http://brugge.med.harvard.edu/protocols>).

**Establishment of cell lines with luciferase activity**

Cells with luciferase activity were used for all *in vivo* metastasis experiments. pSFG-Nes-TGL plasmid containing firefly luciferase was a kind gift from Dr. Ponomarev (Memorial Sloan-Kettering Cancer Center, NY, USA). Retroviral particles containing the plasmid were produced using Amphopack 293 cells and X-tremeGENE HP DNA Transfection Reagent (Roche), following manufacturer’s instructions. 1833 and ANV5 cells were infected with 300 µl of viral particles/well, in the presence of 8 µg/ml polybrene (Sigma-Aldrich) to improve infectiveness. After 48 h, medium was replaced and cells were expanded for Fluorescence Activated Cell Sorting (FACS) of infected cells based on the expression of GFP contained in the pSFG-Nes-TGL plasmid.

**Generation of cells with stable EPCR and SPOCK1 silencing**

Scramble (shControl) and EPCR- and SPOCK1-targeting shRNAs cloned into PLKO.1-puro vector were obtained from Mission^®^ (Sigma-Aldrich), as bacterial glycerol stocks. Plasmid extraction was carried out from grown bacteria with ATP Plasmid Mini Kit (ATP Biotech Inc.) following manufacturer’s instructions. Lentiviral particles were produced using HEK 293T cells, Lentiviral Packaging Mix (Sigma-Aldrich) and X-tremeGENE HP DNA Transfection Reagent (Roche), following manufacturer’s instructions. Cells were infected as detailed for retroviral particles. Puromycin-resistant cells were expanded and frozen. shRNAs used were: shControl (SHC002), shEPCR#1 (TRCN0000061379), shEPCR#2 (TRCN0000061380), shEPCR#3 (TRCN0000323594), shSPOCK1#1 (TRCN0000415564) and shSPOCK1#2 (TRCN0000053595).

Ectopic expression of EPCR and SPOCK1

Empty vector (Control) and EPCR overexpression was performed as described (Antón et al. 2012). Full coding sequence of human SPOCK1 was obtained in pENTRY221 vector (Clone ID 100003544) (GE Dharmacon) and inserted by recombination into pL6-CMV-V5-Dest according to manufacturer´s instructions. After recombination insert was fully sequenced to verify integrity and fidelity.

Total RNA extraction

RNA extraction from cell cultures

Total RNA was extracted from cells seeded at 80% confluence in 28 cm^2^ plates using TRIzol^®^ reagent (GIBCO), following manufacturer’s instructions. RNA concentration and purity were measured in a NanoDrop spectrophotometer, based on absorbance values at 260 and 280 nm. Samples were stored at -80ºC.

RNA extraction from tumors

Frozen tumors obtained at necropsy were crushed using a mortar and pestle, on dry-ice. Crushed frozen pieces were transferred to a 1.5 ml tube with 1 ml of TRIzol^®^ reagent (GIBCO) and homogenized with a T10 Standard Ultra-Turrax (IKA). At this point, RNA extraction was performed as detailed in section 1. For microarray experiments, samples were vigorously mixed and incubated for 3 min at room temperature after the addition of 200 µl of chloroform and centrifuged at 12,000 g for 15 min at 4ºC. Subsequently, RNA extraction was continued using RNeasy Mini Kit (Qiagen) following manufacturer’s specifications. RNA concentration and purity were measured in a NanoDrop spectrophotometer, based on absorbance values at 260 and 280 nm. Samples were stored at -80ºC.

Reverse transcription

RNA was reverse transcribed using DyNAmo cDNA Synthesis Kit (Thermo Scientific). The following mixture was set up for each reaction: 5 µl of buffer, 0.5 µl of random hexamers, 1 µl of transcriptase, 500 ng of RNA and RNase-free water up to 10 µl. Samples were subjected to the following incubation steps in a PTC-100 thermal cycler (MJ Research): 10’ at 25ºC, 30’ at 37ºC and 5’ at 85ºC. cDNA was stored at -20ºC.

**Real-time quantitative PCR (qPCR)**

qPCR was performed in an Applied Biosystems^®^ 7500 Real-Time PCR instrument using FastStart SYBR Green Master (Roche). PCR reactions were performed with 0.5 µl of cDNA (20-25 ng), 0.25 µl of 10 µM forward primer, 0.25 µl of 10 µM reverse primer, 5 µl of FastStart SYBR Green Master and 4 µl of water. Mixes were subjected to the following incubation steps: 2’ at 95ºC, 40 cycles of 15’’ at 95ºC and 1’ at 60ºC and an additional final dissociation step, to calculate Tm of the amplified products in order to evaluate specificity of the reaction. Primer sequences for SPOCK1: 5’-agcacaaggcagaaaggagt (FW) and 5’- cgtggagagctccaaacc (RV). Primer sequences for HPRT: 5'-ggtccttttcaccagcaagct (FW) and 5’-tgacactggcaaaacaatgca (RV).

Data were analyzed using 2^-ΔΔCt^ method for relative gene expression quantification. Gene expression data were normalized with HPRT (ΔCt = Ct target gene – Ct HPRT). All ΔCt values were related to the ΔCt value of the reference sample (shControl): ΔCt sample – ΔCt reference sample.

**Western Blot**

Proteins were extracted when cells reached 80% confluence in 28 cm^2^ dishes. Cells were washed twice with cold DPBS, scraped in protein lysis buffer (1% Nonidet P-40, 150 mM NaCl, 50 mM Tris, 1 mM EDTA, 1 mM phenylmethylsulfonyl fluoride, 1 mM sodium orthovanadate, 5 mM NaF, pH 7.4) supplemented with a protease inhibitor cocktail (Roche) and transferred to 1.5 ml tubes. After 1 h of incubation on ice, lysates were centrifuged for 15 min at 15,000 g at 4ºC, to remove residual cellular debris. Supernatants were transferred to new 1.5 ml tubes and stored at -80ºC. Protein quantification was carried out using Pierce BCA Protein Assay Kit (Thermo Scientific). Proteins (15-30 µg) were denatured at 99ºC for 10’ in 1X loading buffer (32 mM Tris-HCl pH 6.8, 12% glycerol, 2% SDS, 0.005% bromophenol blue and 1 mM DTT). Denatured samples were electrophoresed in discontinuous polyacrylamide gels. Electrophoresis was run under denaturing conditions (SDS-PAGE system) in electrophoresis buffer (25 mM Tris, 192 mM glycine and 0.1% SDS) for 30-60’ at 200 V. Proteins were subsequently transferred to nitrocellulose membranes in transfer buffer (25 mM Tris, 192 mM glycine, 0.05% SDS and 20% methanol) for 2 h at room temperature at 300 mA. Membranes were then blocked in 5% skimmed milk solution (buffered in 0.05% Tween-TBS) for 1 h at room temperature and incubated overnight at 4ºC with primary antibodies. After overnight incubation, blots were washed three times for 5 min with 0.05% Tween-TBS and incubated with horseradish peroxidase (HRP)-linked secondary antibodies at 1:4000 dilution (Amersham) against rabbit, mouse or rat immunoglobulins for 1 h at room temperature. Membranes were washed three times for 5 min with 0.05% Tween-TBS and incubated for 1 min with the peroxidase substrate LumiLightPlus (Roche). Blots were exposed to ECL films (Amersham) for 10-60 s and developed in a Curix 60 processor (AGFA Healthcare).

**Primary and secondary antibodies used for western blot.**

| Protein | 1º Antibody | Manufacturer | Dilution | 2º Antibody |
| --- | --- | --- | --- | --- |
| β-tubulin | H-235 | Santa Cruz | 1:5000 | Anti-rabbit IgG-HRP |
| Human EPCR | 1489 | Dr. Esmon* | 5 µg/ml | Anti-mouse IgG-HRP |
| Murine EPCR | RCR1 | Dr. Fukudome** | 5 µg/ml | Anti-rat IgG-HRP |

* It was kindly gifted by Dr. Charles T. Esmon (Oklahoma Medical Research Foundation, Oklahoma City, USA. ** It was generously provided by Dr. Kenji Fukudome (Saga Medical School, Japan).

**Flow cytometry analysis of immune cells infiltrating mammary tumors**

Cell isolation

Tumors were resected and mechanically dissociated with a razor blade in 3 ml of RPMI, on ice. Minced tumors were transferred to 10 ml of collagenase buffer (0.2% (w/v) collagenase (Sigma-Aldrich) and 4 U/ml DNAse I (Roche) in RPMI) and incubated at 37ºC for 1 h, on a gentle shaker. Digested tumors were centrifuged at 300 g for 8 min at 4ºC. Supernatants were transferred to new tubes and centrifuged again at 300 g for 8 min at 4ºC. Pellets obtained in both centrifugations were resuspended in ice-cold R10 (RPMI + 10% heat-inactivated FBS) and ice-cold DPBS (1:1 ratio) and filtered through 100 µm cell strainers. Samples were centrifuged at 300 g for 8 min at 4ºC and pellets were resuspended and incubated for 2 min at room temperature in 5 ml of Red Blood Lysis Buffer (Sigma-Aldrich). Twenty ml of ice-cold DPBS and R10 (1:1 ratio) were added to the samples prior to centrifugation at 300 g for 6 min at 4ºC. Supernatants were discarded and pellets were resuspended in ice-cold R10 (5-10 ml) and filtered through 100 µm cell strainers before cell counting. Two spleens were subjected to the same procedure and used as controls, except that digestion with collagenase was omitted and the red blood cell lysis step was performed twice.

Cell Staining

Cells were plated in 96-well round-bottom plates at 10^6^ cells/well. Plates were centrifuged at 300 g for 4 min at 4ºC. Supernatant was discarded and cells were resuspended and incubated at 4ºC for 15 min in 50 µl of FACS buffer (1X DPBS, 1% FBS, 0.02% (w/v) sodium azide) with Fc Block™ (BD Biosciences) at 1:50 dilution. Cells were immunostained with antibody solutions for 30 min at 4ºC, in the dark. Cells were washed twice with 150 µl of ice-cold FACS buffer, resuspended in FACS buffer and transferred to test tubes (at a final volume of 500 µl). Ten min before acquisition of the cells, 5 µl of 7AAD (BD Biosciences) were added to the tubes to stain dead cells.

**Antibodies and isotype controls.**

| Antibody | Dilution | Manufacturer | Reference |
| --- | --- | --- | --- |
| CD45-APC | 1:300 | BD Pharmigen | 559864 |
| CD11b-PE | 1:300 | BD Pharmigen | 557397 |
| CD19-APC-eFluor 780 | 1:300 | eBioscience | 47019380 |
| Nkp46-eFluor 450 | 1:100 | eBioscience | 48335180 |
| Ly6G-APC-eFluor 780 | 1:300 | eBioscience | 47593180 |
| Ly6C- PE-Cy7 | 1:300 | BioLegend | 128017 |
| MHCII-eFluor 450 | 1:200 | eBioscience | 48532180 |
| Rat IgG2b-APC | 1:300 | BD Pharmigen | 553991 |
| Rat IgG2c-PE-Cy7 | 1:300 | BioLegend | 400721 |
| Rat IgG1a-eFluor 450 | 1:200 | eBioscience | 48432180 |
| Rat IgG1a-PE | 1:300 | eBioscience | 12432180 |

Note: no isotype control for APC-eFluor 780.

Simultaneously, spleen cells were stained with antibodies (positive control) and with the corresponding isotype controls (negative control) following the same procedure already detailed. OneComp eBeads (BD Biosciences) were used to perform fluorescence compensation, according to manufacturer’s specifications.

Flow cytometry

Cells and beads were acquired in an LSR- II Flow Cytometer (BD Biosciences) in the Flow Cytometry Shared Resource at Cold Spring Harbor Laboratory (NY, USA). FSC/SSC settings and fluorescence detector (PMT) voltages were established with unstained samples and compensations were performed with OneComp eBeads. Around 500,000 cells were acquired per sample. Data were analyzed with FlowJo^®^ software v9.3. 7AAD stained dead cells were excluded from the analysis and cell populations were gated on live cells following the gating strategy shown in Figure S4A.

***In vivo* therapeutic regimen of RCR252**

F(ab’)_2_ RCR252 antibodies were generated by digesting 2 mg of whole-body antibody with 125 µl of pepsin inmobilized on agarose (Pierce) in 20 mM sodium acetate buffer (pH 2.8), at 37ºC in a stirring bath for 1 h. After centrifugation at 1,500 g for 10 min, the supernatant containing F(ab’)_2_ RCR252 was collected with 1 M Tris, pH 9. The efficiency of this process was monitored by SDS-PAGE in non-reducing conditions showing a 110 kDa band. Functional binding of antibodies was demonstrated by Surface Plasmon Resonance (Biacore, GE Healthcare). IgG control antibodies were obtained from Sigma-Aldrich (I4131). Both RCR252 and IgG were resuspended in vehicle (10 mM HEPES, 150 mM NaCl) at 250 µg/ml. Mice were treated with 50 µg of the antibodies daily, administered intraperitoneally in 200 µl of vehicle. Treatment was started the day before the inoculation of cancer cells.

**Histological analyses**

Mammary tumors, lungs and hind limbs were formaldehyde-fixed for 24 h and maintained in 70% ethanol after. Tibiae and femurs were excised and cleaned of all soft tissues before decalcification in Osteosoft solution (Merck) for 72 h, on an orbital shaker. Tissues were embedded in paraffin and cut into 5 µm sections. Slides were dewaxed with xylene and rehydrated through a graded series of ethanol. H&E staining was performed as previously described. For immunohistochemistry, antigen retrieval was carried out and endogenous peroxidase activity was quenched by incubating slides with 3% hydrogen peroxide for 12 min. Sections were incubated with primary antibodies overnight at 4ºC in a humidified chamber. After washing with TBS-Tween, secondary antibodies were applied. Peroxidase activity was revealed with diaminobenzidine (DAB) and sections were lightly counterstained with Harris hematoxylin. Finally, slides were dehydrated in a graded series of ethanol, cleared in xylene and mounted in Cytoseal XYL (Thermo Scientific).

**Antibodies and antigen retrieval conditions used in immunohistochemistry.**

| Antigen | Antibody | AR | Dilution | Raised in | Detection |
| --- | --- | --- | --- | --- | --- |
| Ki67 | Neomarkers (RM9106) | TE, pH 9, 95ºC, 30 min | 1:100 | Rabbit | EnVision anti-rabbit^1^ |
| CD31 | Dianova  (DIA 310) | TE, pH 9, 95ºC, 30 min | 1:50 | Rat | Rabbit anti-rat^2^  EnVision anti-rabbit^1^ |
| Caspase-3 | Cell Signaling (9661) | TE, pH 9, 95ºC, 30 min | 1:100 | Rabbit | EnVision anti-rabbit^1^ |
| F4/80 | eBiosciences (14-4801-82) | PK 20 µg/ml, 37ºC, 30 min | 1:400 | Rat | Rabbit anti-rat^2^  EnVision anti-rabbit^1^ |
| EPCR | HEPCR1489* | TE, pH 9, 95ºC, 30 min | 1:200 | Mouse | EnVision anti-mouse^3^ |

*This monoclonal antibody (1.5 mg/ml) was produced and kindly provided by Dr. Charles Esmon. AR: Antigen retrieval. TE: 10 mM Tris, 1 mM EDTA. PK: Proteinase K. ^1^Dako K4011, ^2^Dako E0468 (1:200), ^3^Dako K4007.

All slides were captured using a Zeiss Axioimager M1 (Zeiss) microscope and ZEN (Zeiss) software. H&E-stained slides were captured at 2.5X magnification. Tumor area was calculated using Fiji software, by manually delineating tumor and total bone or lung areas. For immunohistochemical analyss, random pictures (5-25, depending on tumor size for each tumor) were taken at 20 X magnification and stained areas or cells were quantified using Fiji software.
