# Supplementary figures and images for: EPCR promotes breast cancer progression by altering SPOCK1/testican 1-mediated 3D growth
Source: J Hematol Oncol. 2017 Jan 19;10:23. doi: 10.1186/s13045-017-0399-x (PMC5248526; doi:10.1186/s13045-017-0399-x)

## Slide 1
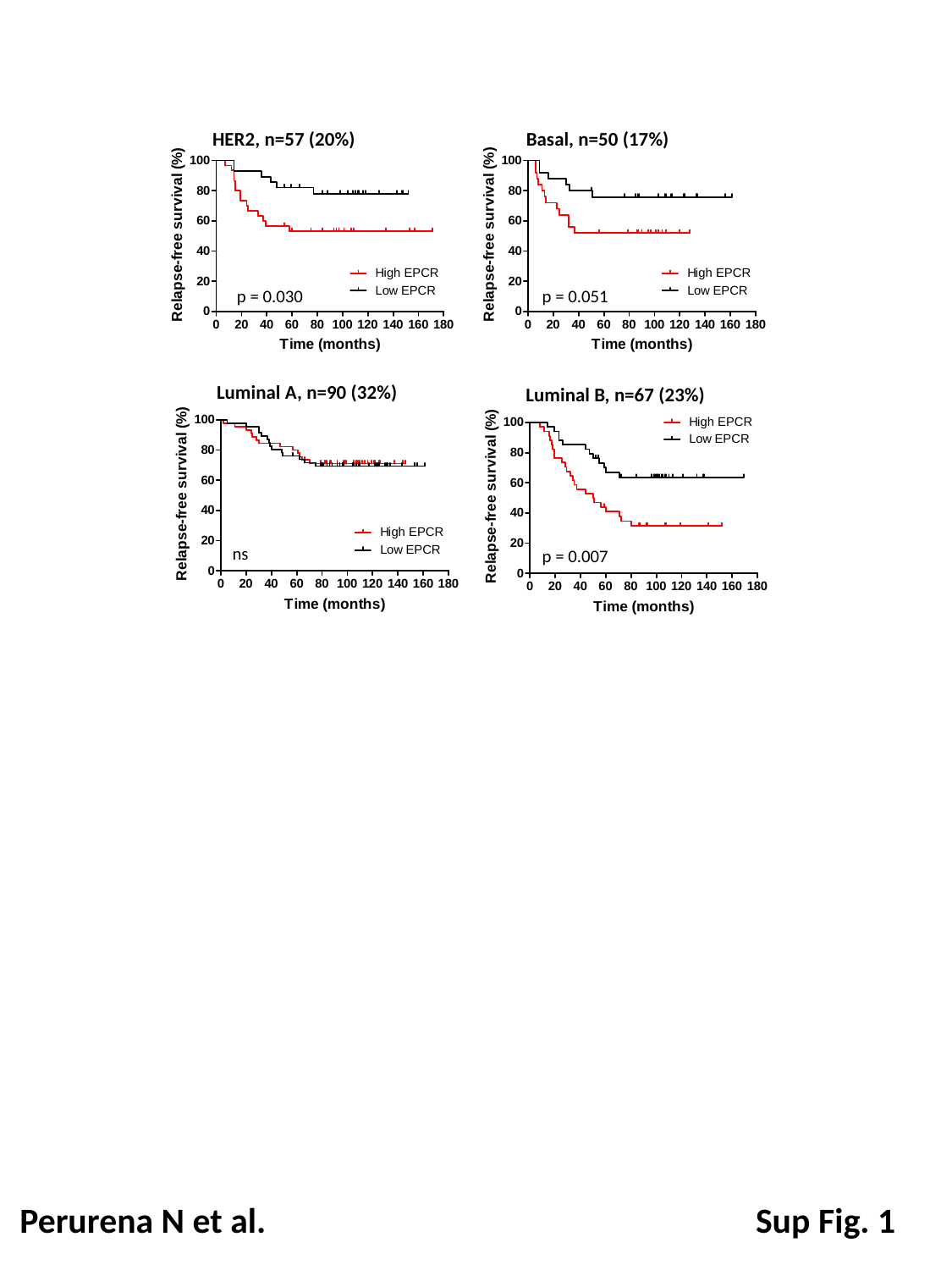

HER2, n=57 (20%)
Basal, n=50 (17%)
Luminal A, n=90 (32%)
Luminal B, n=67 (23%)
p = 0.051
p = 0.030
ns
p = 0.007
Perurena N et al.
Sup Fig. 1

Supplement: Supplementary file 2 — Kaplan–Meier analysis in different molecular subtypes of breast cancer patients based on EPCR expression levels. Relapse-free survival curves for each molecular subtype of breast cancer. Log-rank test was used to determine p values in all cases. (PPTX 273 kb) [file 13045_2017_399_MOESM2_ESM.pptx]

## Slide 1
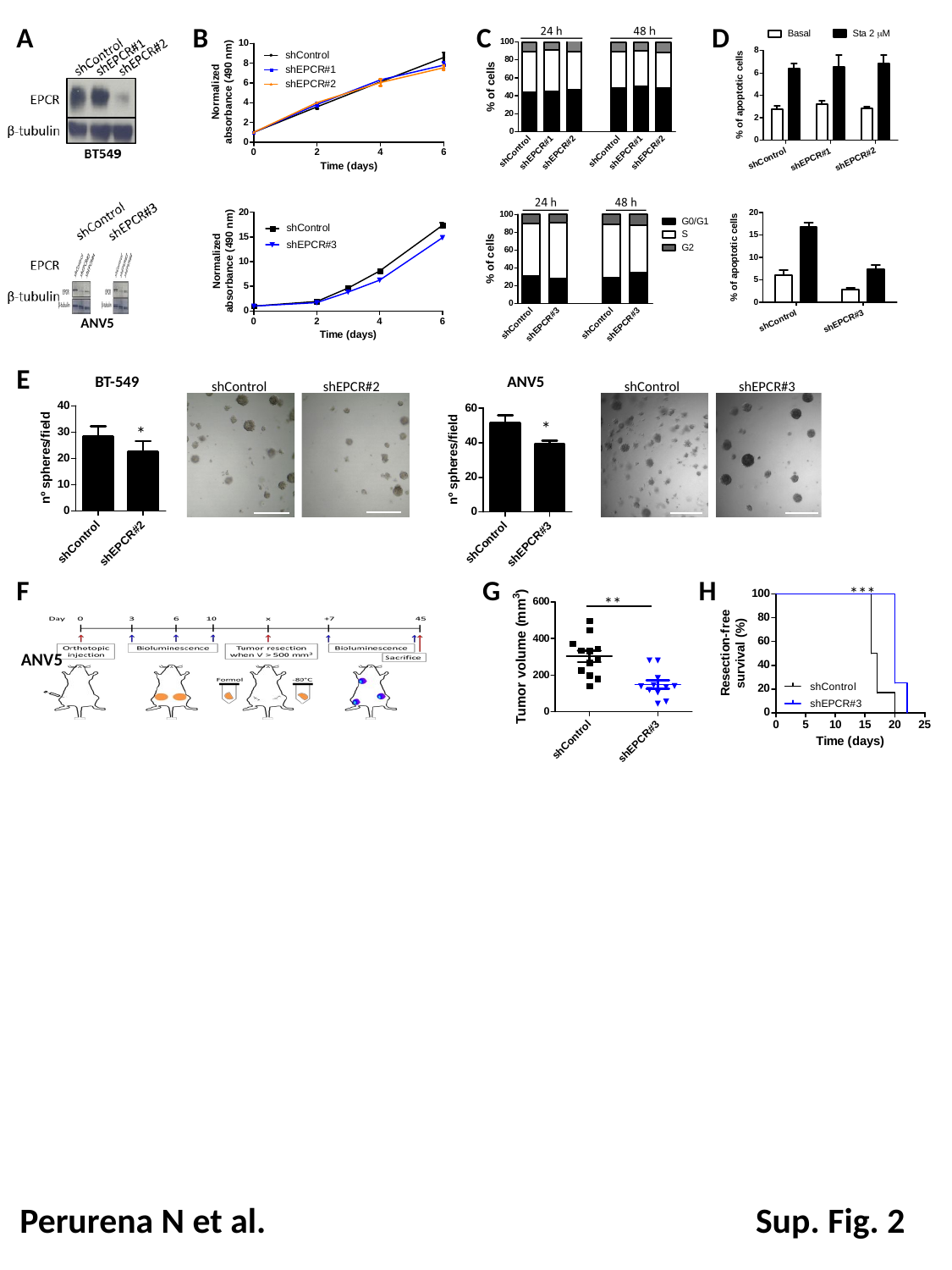

B
A
C
D
ANV5
E
BT-549
*
ANV5
*
shControl
shEPCR#2
shControl
shEPCR#3
F
***
**
ANV5
G
H
Perurena N et al.
Sup. Fig. 2

Supplement: Supplementary file 3 — Effects of EPCR silencing in vitro and in vivo tumor growth in an orthotopic model. A. Western blot analysis of EPCR protein levels in human BT-549 (top) and murine ANV5 (bottom) cells transduced with a scramble shRNA (shControl) and shRNAs targeting human (shEPCR#1 and shEPCR#2 in BT-549) and murine (shEPCR#3 in ANV5) EPCR. β-tubulin was used as loading control. White line indicates that the membrane was cut. B. MTS in vitro proliferation assay of BT-549 (top) and ANV5 (bottom) cells. Data were normalized with absorbance values from day 0 and represent mean ± SD of six replicates. C. Percentage of BT549 (top) and ANV5 (bottom) cells in each phase of the cell cycle after maintaining cells in culture for 24 and 48 h. Sta, staurosporine. D. Percentage of apoptotic BT-549 (top) and ANV5 (bottom) cells in basal and staurosporine-induced conditions, measured by annexin-V binding flow cytometry assay. E. Quantification of spheres grown in 3D matrigel cultures. Data are mean ± SD of 8 replicates. Representative images at ×4 magnification. Scale bar 0.5 mm. F. Outline of the in vivo orthotopic experiment (n = 8 per group). G. Quantification of tumor volume at day 15 post-injection. H. Kaplan–Meier curves of resection-free survival. (PPTX 9850 kb) [file 13045_2017_399_MOESM3_ESM.pptx]

## Slide 1
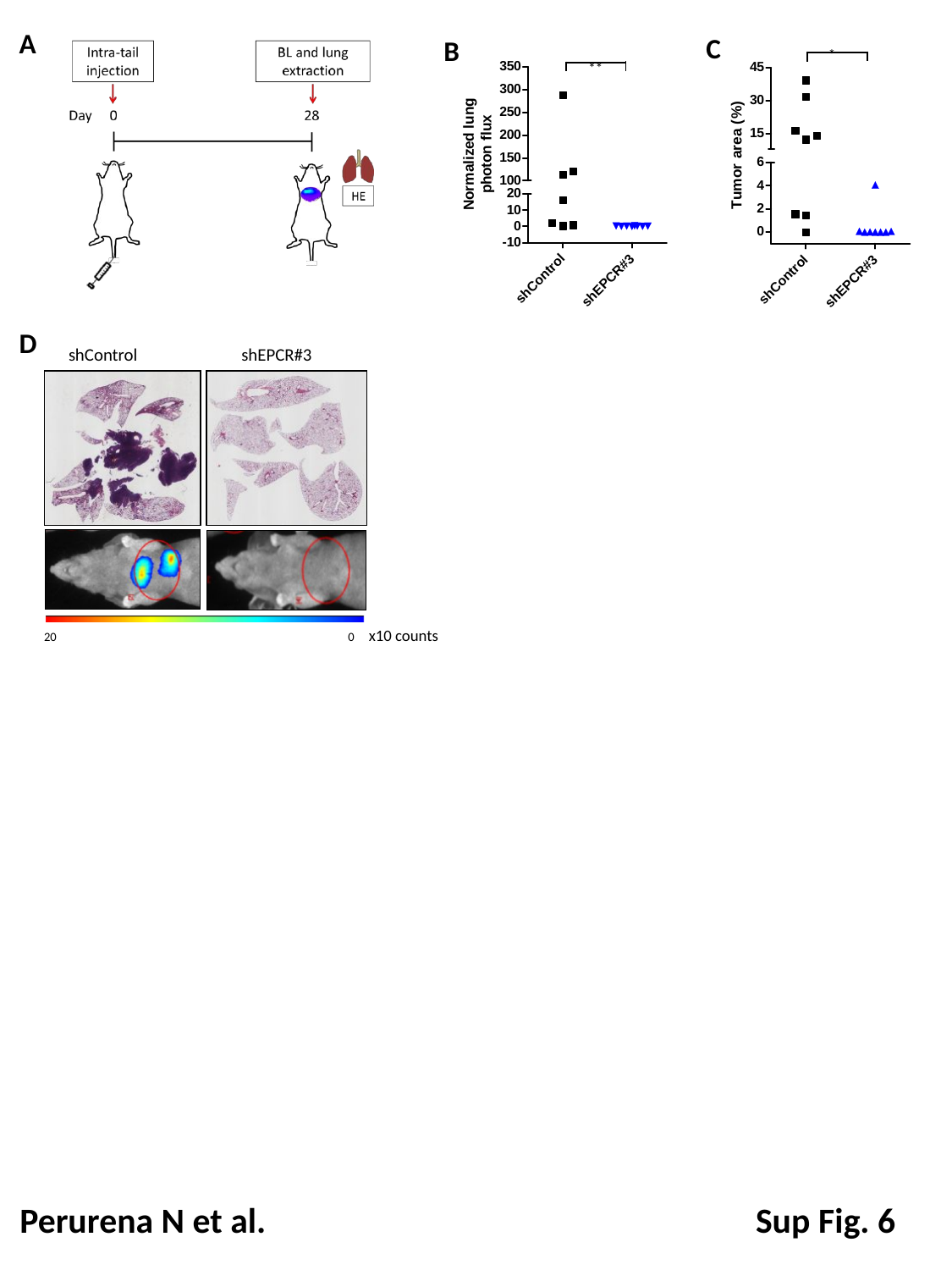

A
C
B
**
D
shEPCR#3
shControl
 0 x10 counts
*
Perurena N et al.
Sup Fig. 6

Supplement: Supplementary file 7 — Effects of EPCR silencing in the ability of murine ANV5 cells to metastatize to the lungs. A. Outline of the intra-tail injection experiment (n = 8 per group). Quantification of bioluminescence signals (B) and tumor area (C) in the lungs at the end of the experimental period (day 28 post-injection). Each dot represents one mouse. D. Representative images of H&E-stained lung sections (top) and BLI (bottom). *p < 0.05, **p < 0.01. (PPTX 1080 kb) [file 13045_2017_399_MOESM7_ESM.pptx]

## Slide 1
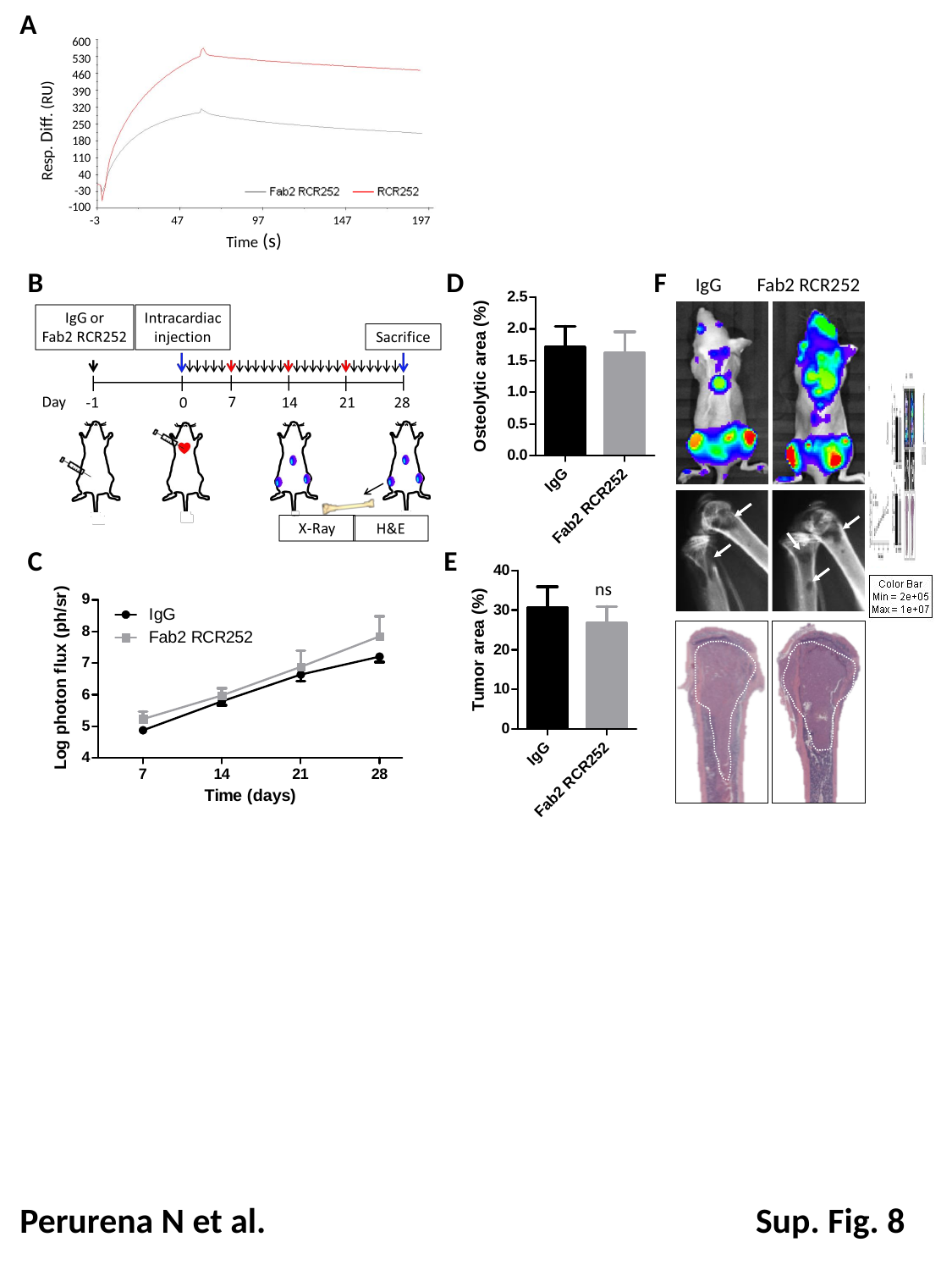

A
600
530
460
390
320
250
180
110
40
-30
-100
Resp. Diff. (RU)
-3 47 97 147 197
Time (s)
B
D
F
IgG Fab2 RCR252
C
E
ns
Perurena N et al.
Sup. Fig. 8

Supplement: Supplementary file 9 — Effects of the pharmacological EPCR blockade in the prometastatic activity of 1833 cells. A. Specificity of anti-EPCR antibodies, RCR252, and its F(ab´)2 fraction, by surface plasmon resonance (SPR). EPCR (500 RU) was immobilized through the anti-EPCR antibody RCR2 (that does not bind in the ligand-receptor domain) on a CM5 chip. The binding of 250 nM of RCR252 and its F(ab´)2 fraction to the EPCR were monitored. A representative experiment is shown. RU resonance units; s, seconds. B. Outline of the experiment (n = 8 per group). C. Photon flux quantification in hind limbs. D. Tumor area quantification in H&E-stained bone sections. E. Osteolytic bone area quantification in X-ray images from day 28 post-injection. F. Representative images of BLI (top), X-rays (middle), and H&E staining (bottom) at day 28 post-injection. All data are represented by mean ± SEM. (PPTX 1540 kb) [file 13045_2017_399_MOESM9_ESM.pptx]

## Slide 1
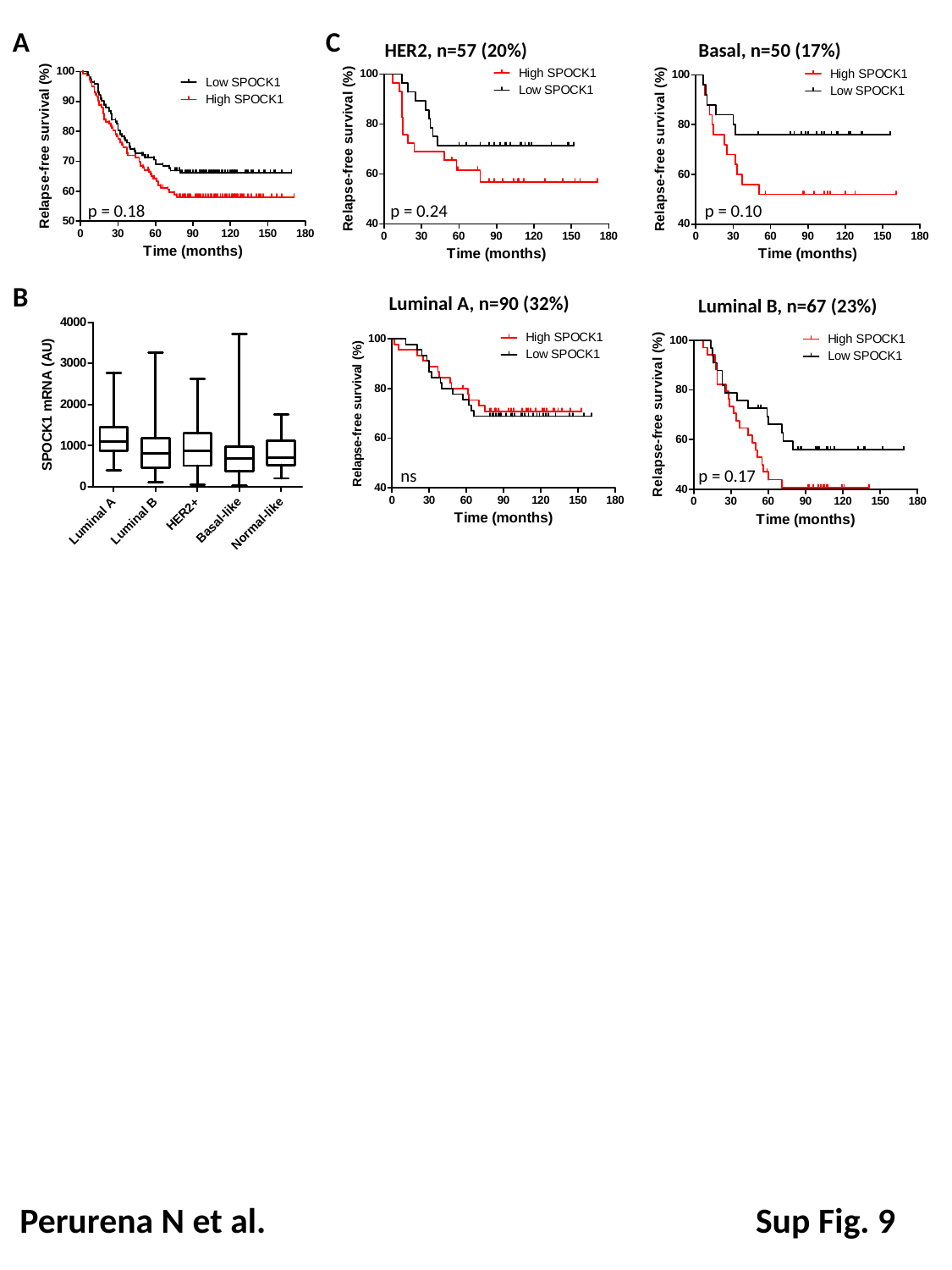

A
p = 0.18
C
HER2, n=57 (20%)
Basal, n=50 (17%)
Luminal A, n=90 (32%)
Luminal B, n=67 (23%)
p = 0.24
p = 0.10
ns
p = 0.17
B
Perurena N et al.
Sup Fig. 9

Supplement: Supplementary file 10 — Clinical relevance of SPOCK1 in different breast cancer subtypes. A. Relapse-free survival analysis of all patients included in the GSE2034 cohort (n = 286), classified into “high SPOCK1” and “low SPOCK1” based on median expression value of SPOCK1. B. SPOCK1 mRNA expression levels in the primary tumors, classified by molecular subtypes. Whiskers represent minimum and maximum values. AU, arbitrary units. C. Relapse-free survival curves for each molecular subtype of breast cancer. Log-rank test was used to determine p values in all cases. ns, non-statistical significance. (PPTX 288 kb) [file 13045_2017_399_MOESM10_ESM.pptx]

## Slide 1
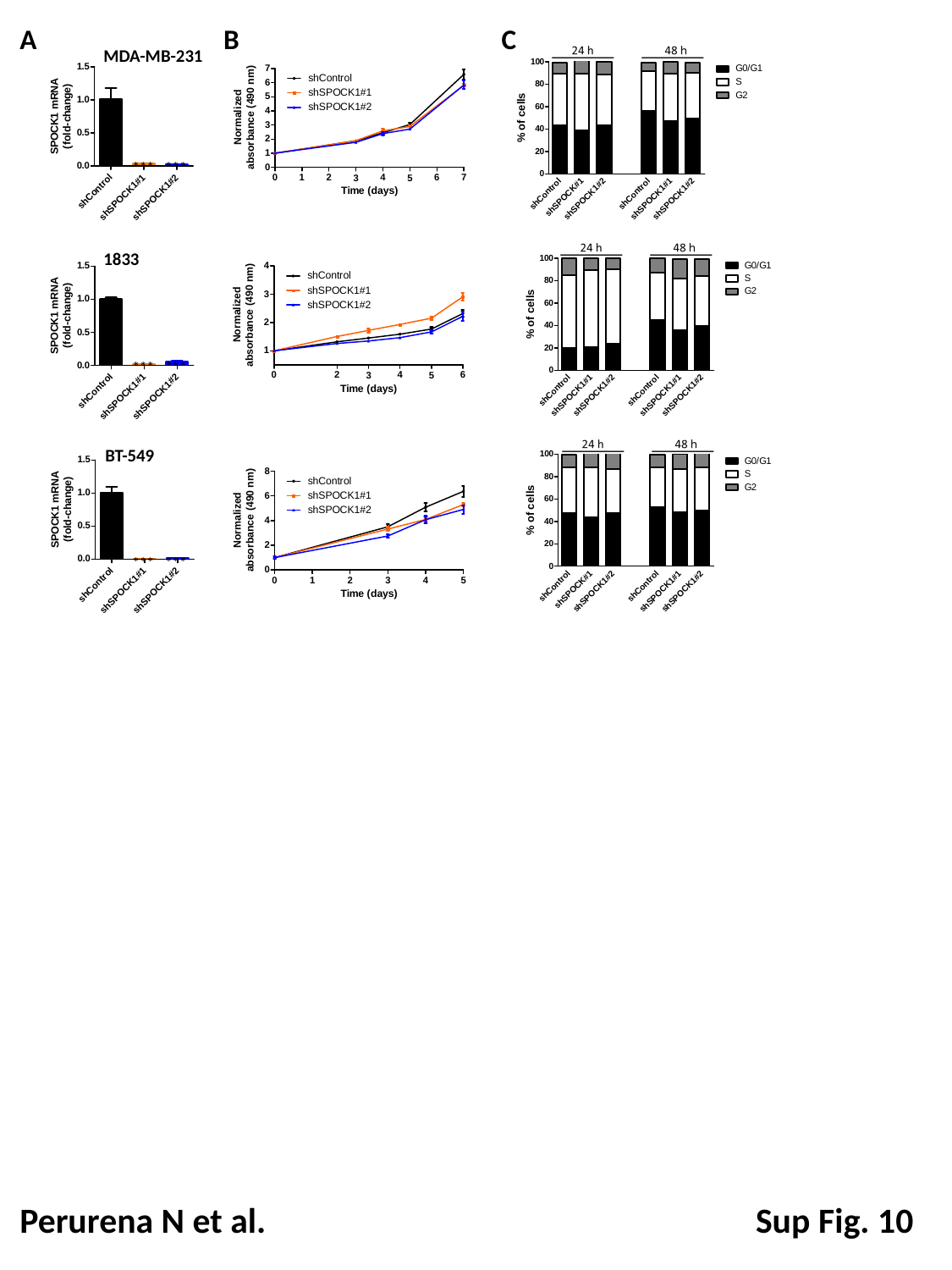

B
C
A
BT-549
1833
MDA-MB-231
***
***
***
***
***
***
Perurena N et al.
Sup Fig. 10

Supplement: Supplementary file 11 — Cell growth kinetics in 2D cultures of human breast cancer cell lines after SPOCK1 silencing. A. Analysis of SPOCK1 expression levels by RT-qPCR in human cells transduced with a scramble shRNA (shControl) and two different shRNAs (shSPOCK#1 and shSPOCK#2) targeting human SPOCK1. B. MTS in vitro proliferation assay of MDA-MB-231 (top), 1833 (middle), and BT549 (bottom) cells. Data were normalized with absorbance values from day 0 and represent mean ± SD of six replicates. Experiments were repeated three times with similar results. C. Percentage of MDA-MB-231 (top), 1833 (middle), and BT549 (bottom) cells in each phase of the cell cycle, after maintaining cells in culture for 24 and 48 h. (PPTX 223 kb) [file 13045_2017_399_MOESM11_ESM.pptx]
