# Supplementary material for: EPCR promotes breast cancer progression by altering SPOCK1/testican 1-mediated 3D growth
Source: J Hematol Oncol. 2017 Jan 19;10:23. doi: 10.1186/s13045-017-0399-x (PMC5248526; doi:10.1186/s13045-017-0399-x)
Supplement: Supplementary file 4 — Immunohistochemical analysis of several markers in control and EPCR-silenced size-matched mammary tumors resected at different time points. A. Representative images showing H&E staining (×2.5 magnification) and the immunohistochemical staining of Ki67, cleaved caspase-3, CD31, and F4/80 (×20 magnification) in formaldehyde-fixed tumors. Scale bars 80 μm (H&E) and 10 μm (Ki67, caspase-3, CD31, and F4/80). T. mass, tumor mass. T. border, tumor border. B. Quantification of the percentage of immunoreactive cells. Each dot represents one tumor. Data are mean ± SEM. ns means non-statistical significance. (PPTX 2780 kb) [file 13045_2017_399_MOESM4_ESM.pptx]

## Slide 1
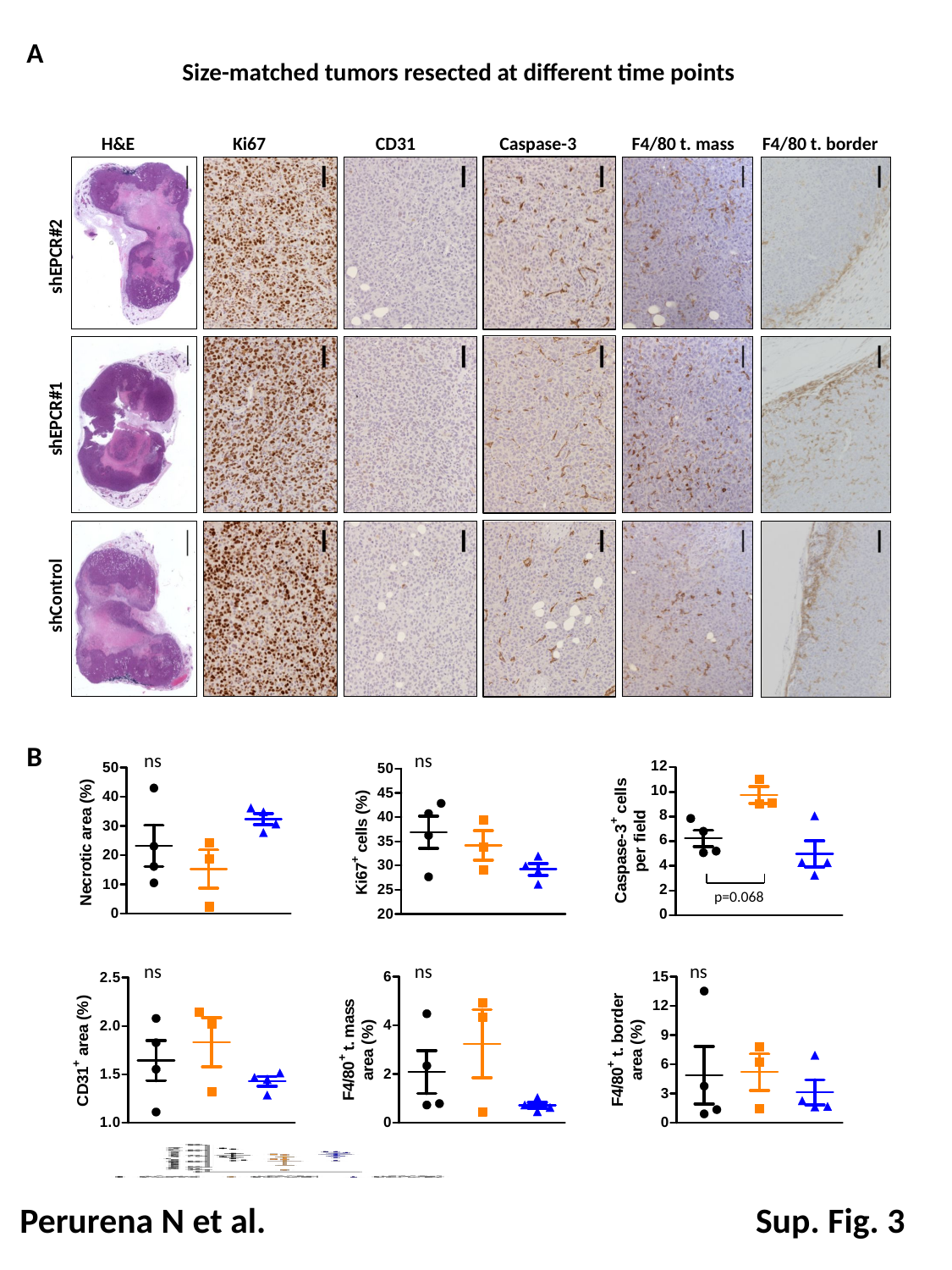

shControl
shEPCR#1
shEPCR#2
H&E
Ki67
CD31
Caspase-3
F4/80 t. mass
F4/80 t. border
A
Size-matched tumors resected at different time points
B
ns
ns
ns
ns
ns
p=0.068
Perurena N et al.
Sup. Fig. 3
