# Supplementary material for: EPCR promotes breast cancer progression by altering SPOCK1/testican 1-mediated 3D growth
Source: J Hematol Oncol. 2017 Jan 19;10:23. doi: 10.1186/s13045-017-0399-x (PMC5248526; doi:10.1186/s13045-017-0399-x)
Supplement: Supplementary file 5 — Effects of EPCR silencing in cell growth kinetics and immune infiltration of control and EPCR-silenced mammary tumors resected at the same time point. A. Outline of the experiment (n = 5 per group). B. Tumor volume at the end of the experimental period (day 32 post-injection). Each dot represents one tumor. Data are mean ± SEM. C. Representative images showing H&E staining (×2.5 magnification) and the immunohistochemical staining of Ki67, cleaved caspase-3, CD31, and F4/80 (×20 magnification). T. mass, tumor mass. T. border, tumor border. Scale bars 80 μm (H&E) and 10 μm (Ki67, caspase-3, CD31, and F4/80). D. Quantification of the percentage of immunoreactive cells. Each dot represents one tumor. Data are mean ± SEM. *p < 0.05. ns means non-statistical significance. (PPTX 2260 kb) [file 13045_2017_399_MOESM5_ESM.pptx]

## Slide 1
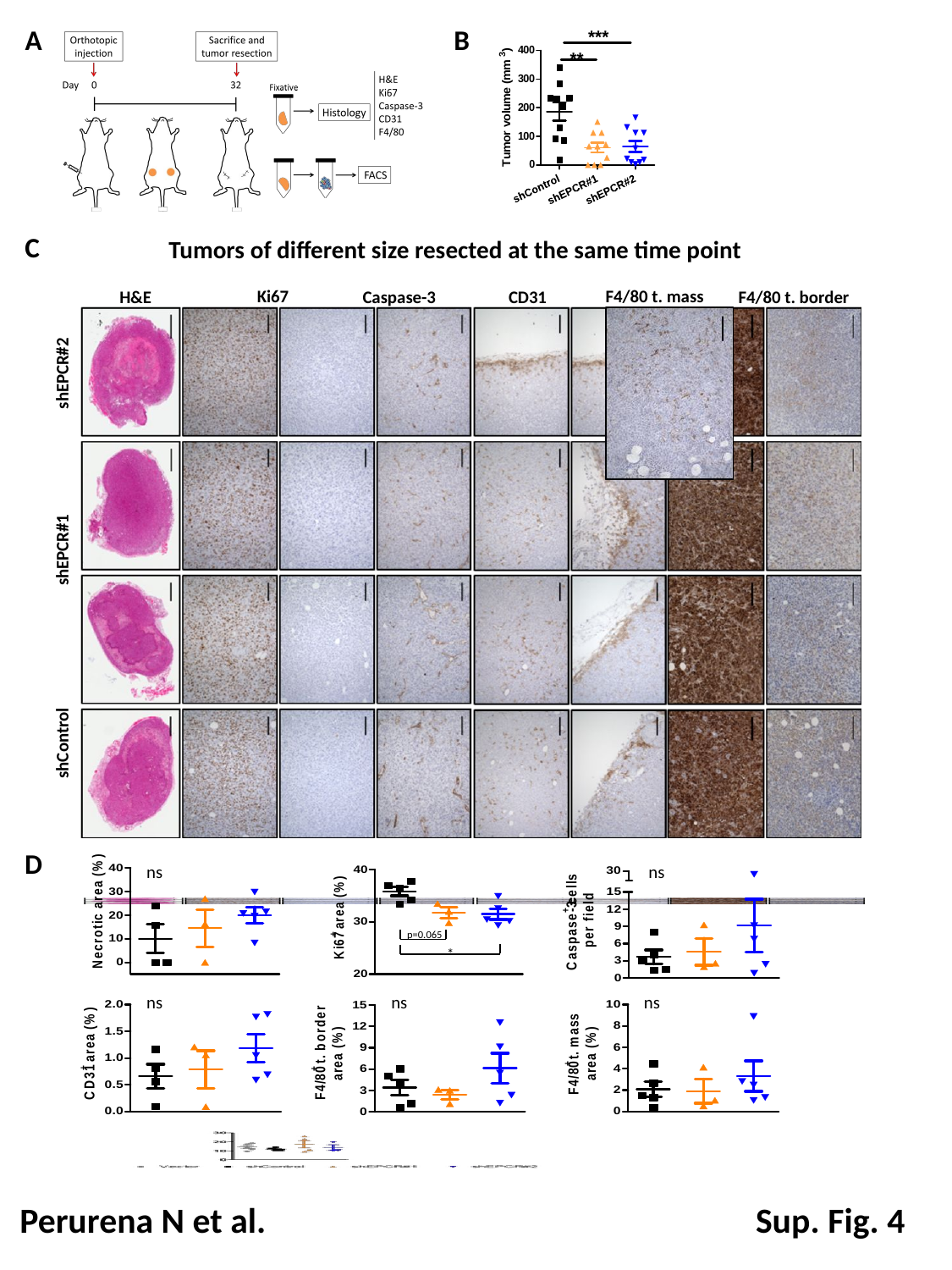

A
B
C
Tumors of different size resected at the same time point
shEPCR#2
shControl
shEPCR#1
H&E
Ki67
Caspase-3
CD31
F4/80 t. mass
F4/80 t. border
D
ns
ns
ns
ns
ns
*
p=0.065
Perurena N et al.
Sup. Fig. 4
