# Supplementary material for: EPCR promotes breast cancer progression by altering SPOCK1/testican 1-mediated 3D growth
Source: J Hematol Oncol. 2017 Jan 19;10:23. doi: 10.1186/s13045-017-0399-x (PMC5248526; doi:10.1186/s13045-017-0399-x)
Supplement: Supplementary file 6 — Analysis of immune cells infiltrating control and EPCR-silenced mammary tumors. A. Flow cytometry gating strategy. Arrows of the same color indicate simultaneous detection of markers. MDSCs, myeloid derived suppressor cells. NK, natural killer cells. DCs, dendritic cells. B. Quantification of the percentage of immune subpopulations infiltrating the tumors. Each dot represents one tumor. Data are mean ± SEM. (PPTX 932 kb) [file 13045_2017_399_MOESM6_ESM.pptx]

## Slide 1
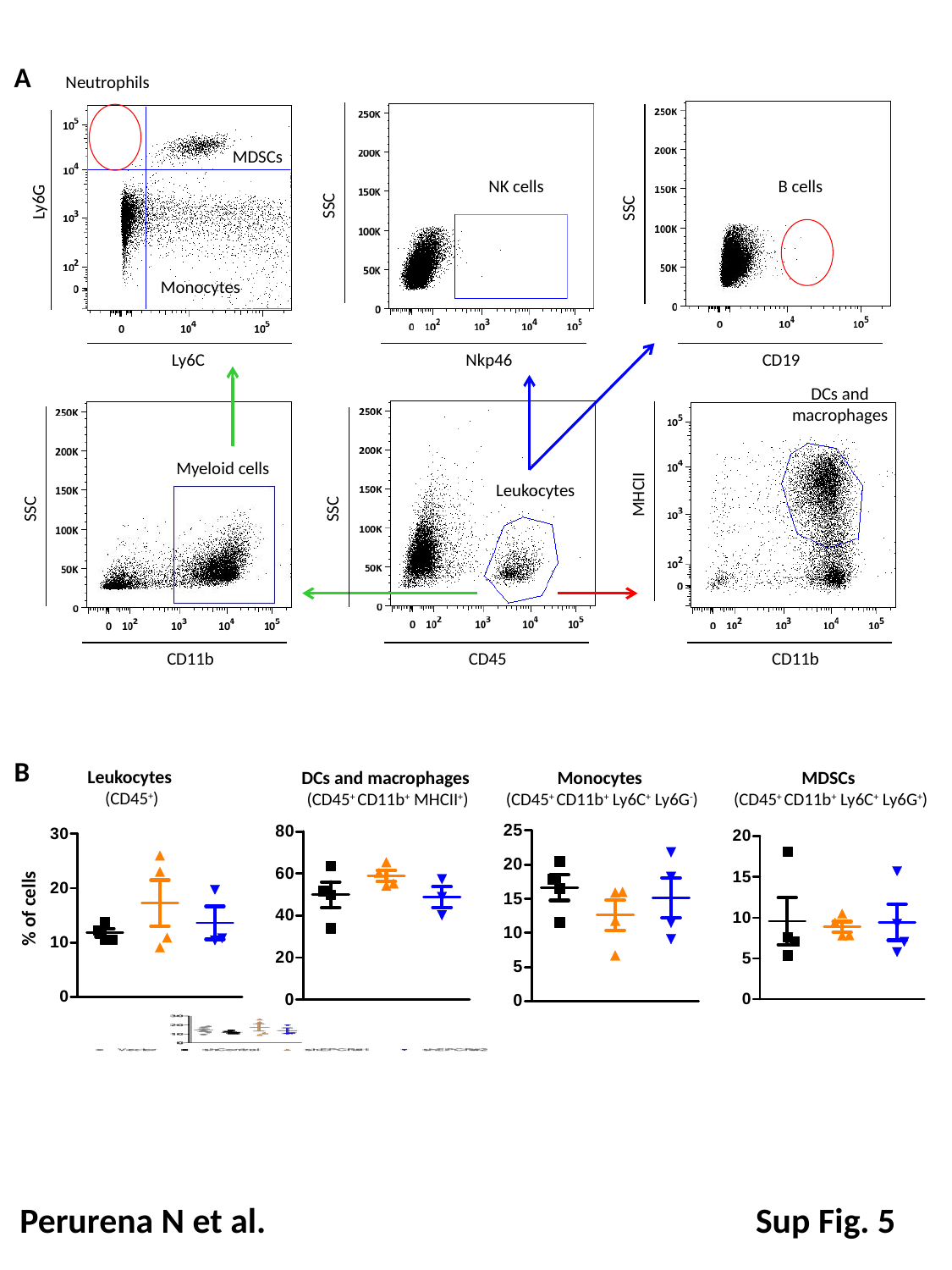

A
Ly6G
Ly6C
Neutrophils
MDSCs
B cells
NK cells
Monocytes
DCs and macrophages
Myeloid cells
Leukocytes
B
Leukocytes
(CD45+)
Monocytes
(CD45+ CD11b+ Ly6C+ Ly6G-)
MDSCs
(CD45+ CD11b+ Ly6C+ Ly6G+)
DCs and macrophages
(CD45+ CD11b+ MHCII+)
% of cells
SSC
SSC
CD19
Nkp46
MHCII
SSC
SSC
CD11b
CD45
CD11b
Perurena N et al.
Sup Fig. 5
