# Supplementary material for: EPCR promotes breast cancer progression by altering SPOCK1/testican 1-mediated 3D growth
Source: J Hematol Oncol. 2017 Jan 19;10:23. doi: 10.1186/s13045-017-0399-x (PMC5248526; doi:10.1186/s13045-017-0399-x)
Supplement: Supplementary file 8 — Cell growth kinetics of APC-stimulated breast cancer cell lines. A. MTS proliferation assay of cells stimulated with increasing doses of APC. Data were normalized with absorbance values from day 0. Each dot represents mean ± SD of six replicates. B. Percentage of cells in each phase of the cell cycle in control and 50 nM APC-stimulated cells for 24 and 48 h, in serum-free and 4% serum medium. C. Percentage of apoptotic cells in basal and staurosporine-induced conditions, measured by annexin-V binding flow cytometry assay. Cell lines are MDA-MB-231,1833, BT-549, and ANV5, from the left to the right, in all figure sections. (PPTX 325 kb) [file 13045_2017_399_MOESM8_ESM.pptx]

## Slide 1
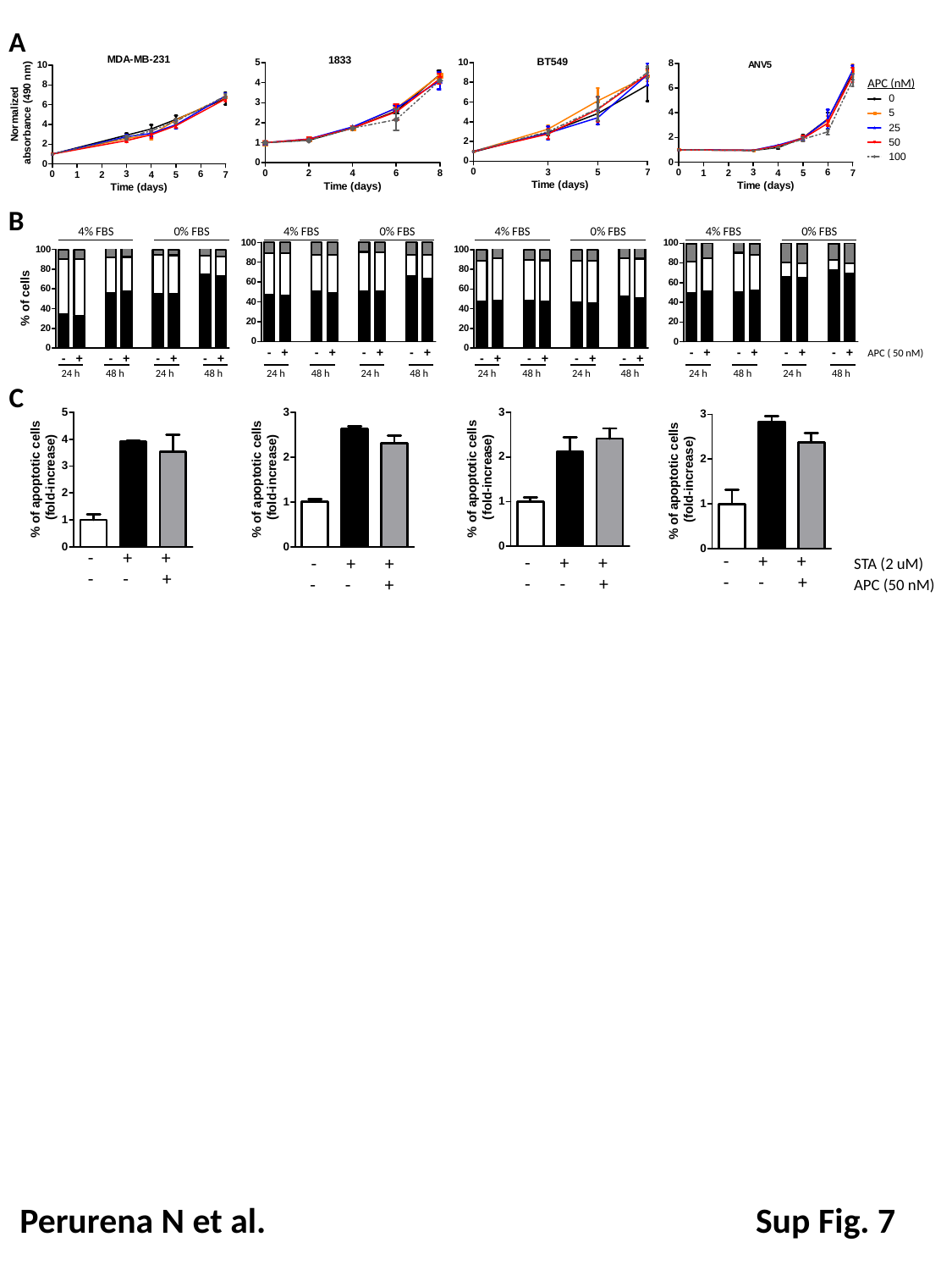

A
APC (nM)
B
4% FBS
0% FBS
APC ( 50 nM)
24 h
48 h
24 h
48 h
4% FBS
0% FBS
24 h
48 h
24 h
48 h
4% FBS
0% FBS
24 h
48 h
24 h
48 h
4% FBS
0% FBS
24 h
48 h
24 h
48 h
C
- + +
- - +
- + +
- - +
- + +
- - +
- + +
- - +
STA (2 uM)
APC (50 nM)
Perurena N et al.
Sup Fig. 7
